# The Therapeutic Effects of Adipose-Derived Stem Cells and Recombinant Peptide Pieces on Mouse Model of DSS Colitis

**DOI:** 10.1177/0963689718782442

**Published:** 2018-07-06

**Authors:** Reiko Iwazawa, Sayako Kozakai, Tsukasa Kitahashi, Kentaro Nakamura, Ken-ichiro Hata

**Affiliations:** 1 Regenerative Medicine Research Laboratories, FUJIFILM Corporation, Kanagawa, Japan

**Keywords:** adipose-derived stem cells, inflammatory bowel disease, veterinary therapy, dextran sulfate sodium-induced colitis mouse

## Abstract

Cell therapies using adipose-derived stem cells (ADSCs) have been used to treat inflammatory bowel disease (IBD) in human and dog. We previously reported the CellSaic technique, which uses a recombinant scaffold to enhance the efficacy of cell therapy. To examine whether this technique can be applied to cell therapy for colitis, we evaluated the efficacy of CellSaic in colitis mouse models. Colitis mouse models were developed by administering dextran sulfate sodium (DSS) to C57BL/6 mice for 7 days. Then CellSaic comprising human/canine ADSCs (1.2 × 10^6^ cells) or human/canine ADSCs only (1.2 × 10^6^ cells) were administered to the mice. The body weights were measured, and the colon length measurements and histological evaluations were conducted at 7 days after administration. After *in vitro* culture of human ADSC (hADSC) CellSaic and hADSC spheroids in medium containing TNFα, the levels of the anti-inflammatory protein TSG-6 in each supernatant were measured. Furthermore, we conducted tumorigenicity and general toxicity tests of canine ADSC (cADSC) CellSaic in NOG mice for 8 weeks. In the colitis mouse models, the ADSC CellSaic group presented recovery of body weight and colon length compared with the ADSC-only group. Histological analysis showed that ADSC CellSaic decreased the number of inflammatory cells and repaired ulceration. *In vitro*, hADSC CellSaic secreted 3.1-fold more TSG-6 than the hADSCs. In addition, tumorigenicity and general toxicity of cADSC CellSaic were not observed. This study suggests that human and canine ADSC CellSaic has a therapeutic effect of colitis in human and dogs.

## Introduction

Human and veterinary therapies are considered to be integrated. Synergy between veterinarians, physicians, and other scientific health and environmental professionals has been promoted in an initiative known as “One Health,” which is meant to improve the lives of all species by integrating human and veterinary medicine^1^. The “One Health” initiative aims to efficiently transform medicine, particularly the field of regenerative medicine, for all species. Moreover, the role of veterinary patients in the evolution of stem cell therapies for both human and animal patients will be explored^2–5^.

Stem cell therapies for both human and animals have been studied for regenerative medicine, mainly adipose-derived stem cells (ADSCs) and bone marrow mesenchymal stem cells (BMSCs). There are numerous reports of cell therapies using ADSCs and BMSCs in humans^6–8^. In veterinary regenerative medicine, autologous ADSC therapy has been commercially available since 2003^9^. Previously reported results from a blinded, controlled trial in dogs with chronic osteoarthritis of the coxofemoral joint demonstrated efficacy of a single intraarticular injection of autologous ADSC therapy^10^.

Therapies using ADSCs and BMSCs are beneficial for treating inflammatory bowel disease (IBD), a chronic relapsing disease in which pro-inflammatory immune cells and cytokines induce intestinal tissue damage and disability^11–14^. In a clinical study of humans, more than 200 patients were treated by local injections of mesenchymal stem cells (MSCs), resulting in a complete response in more than half of the patients and overall response in approximately two-thirds of the patients^11^. In a clinical study of dogs, 11 dogs with confirmed IBD received one intravascular infusion of MSCs. Clinical remission occurred in 9 of the 11 dogs at day 42, while the 2 remaining dogs demonstrated a partial response^15^.

Wang et al. examined intravenous injection (i.v.), intraperitoneal injection (i.p.), and anal injection (a.i.) as methods for administering BMSCs. They found that i.p. leads to a better recovery from colitis and may be the optimum BMSC delivery route for treating dextran sulfate sodium (DSS)-induced colitis^16^. Chen et al. reported that interferon BMSCs, which express interferon-γ, more efficiently ameliorated DSS-induced colitis in mice, showing regained colitis-related loss of body weight, increased colon length, decreased disease activity index, and improved tissue structure of the small intestine^17^. As for other therapies using MSC for colitis, especially cell-free therapies, Legaki et al. have reported that conditioned media from MSC ameliorate DSS-induced colitis in an immunodeficient mouse model^18^. Therefore, paracrine effects by secreted molecules from MSC may have the therapeutic effects on DSS-induced mice.

In this study, we considered using a scaffold as an approach to therapy. Scaffolds are important for adherent cells because apoptosis is induced by lack of cell/scaffold attachment^19,20^. We previously reported a new cell transplantation platform, CellSaic, derived from cell- and scaffold-forming mosaic, and using a bioabsorbable biomaterial known as recombinant protein (RCP), which was developed by the FUJIFILM Corporation (Tokyo, Japan). We confirmed that cell viability in the BMSC CellSaic was higher than that in the spheroid *in vitro*. Furthermore, cells in the implanted CellSaic showed high survival rates, with blood vessels easily forming in the grafts compared with cells only. CellSaic can prevent graft cell death in spheroids by using petaloid pieces of RCP^21^.

As described above for “One Health,” we also considered that the clinical study of CellSaic for dogs (companion animals) would enhance the development of these methods for humans. Therefore, we evaluated the efficacy of the CellSaic technique in mouse models of colitis using human or canine ADSCs. We assume this study as a step forward to prove advantages of scaffolds in treatment of disease models *in vivo*. We also evaluated the safety, tumorigenicity, and general toxicity of canine ADSC (cADSC) CellSaic by subcutaneous implantation of the cells into NOG mice. This study may provide a new colitis therapy for both humans and dogs.

## Materials and Methods

### Materials

Human ADSCs (hADSCs) were purchased from Lonza (PT-5006, Basel, Switzerland). The medium for hADSC was Bulletkit ADSC (PT-4505, Lonza). cADSCs were generated as previously described^22^. Briefly, adipose tissue from a TOYO beagle dog (female, 12 months old) was cut into small pieces and digested at 37°C for 1 h with 2 mg/mL collagenase (Nitta gelatin, Tokyo, Japan) in Dulbecco’s phosphate-buffered saline. The sample was centrifuged at 600 ×*g* for 5 min, washed, centrifuged again, and seeded into a flask containing MSCGM (PT-2501, Lonza,). The medium used for cADSCs was high-glucose DMEM (043-30085, Wako, Osaka, Japan) containing 10% fetal bovine serum (30-2020, ATCC, Manassas, VA, USA), and penicillin streptomycin/amphotericin B (161-23181, Wako). RCP pieces were generated as previously described and we used the petaloid pieces^18^. C57BL/6 N mice (5 weeks old, female) were obtained from Charles River Japan, Inc. (Yokohama, Japan).

### Formation of hADSC/cADSC CellSaic Platforms

hADSCs were cultured at 37°C in a 5% CO_2_ humidified atmosphere. CellSaic platforms, containing hADSCs, were prepared by mixing hADSCs (3 × 10^5^ cells/mL) and RCP pieces (0.25 mg/mL) in Bulletkit ADSC medium; this mixture was seeded on a 35-mm dish of EZ SPHERE 4000-903SP (AGC Techno Glass, Haibara, Japan).

For cADSC CellSaic formation, hADSCs in Bulletkit ADSC medium were prepared by mixing 3 × 10^5^ cells/mL and RCP pieces (0.0625 mg/mL). The mixture was then seeded onto an EZ SPHERE 4000-903SP. Each dish was incubated for 72 h. hADSC and cADSC CellSaics were collected into 400 µL of the respective medium and used for implantation.

### Efficacy test (Fig. 1A)

#### Creation of the colitis model

Acute colitis was induced in C57BL/6 female mice by feeding of 3% DSS (MP Biomedicals, Santa Ana, CA, USA) dissolved in water for 7 days, followed by 7 days of regular drinking water.

**Fig. 1.**
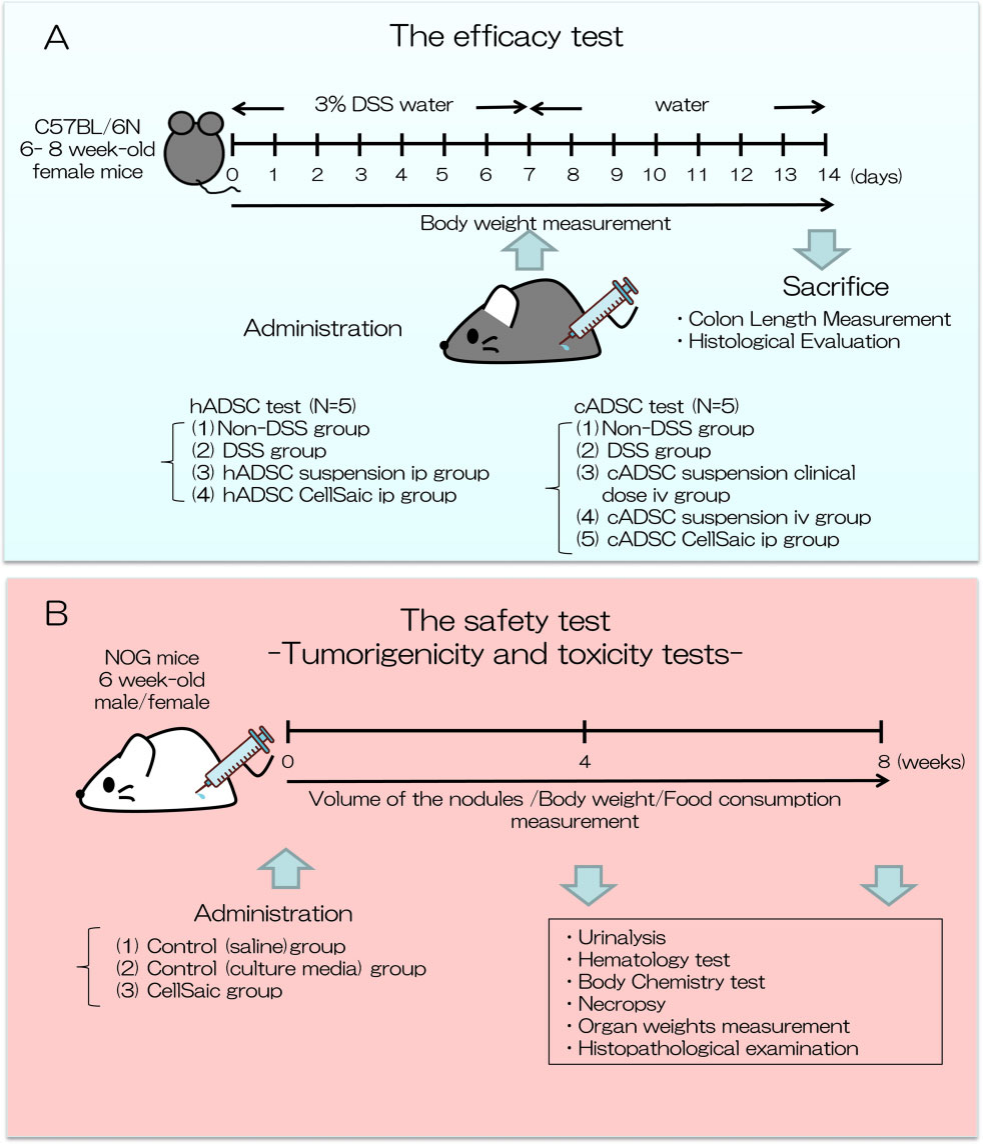
Experimental design of this study. We conducted an efficacy test and safety test of ADSC CellSaic. (A) Efficacy test: therapeutic effects of hADSC/cADSC CellSaic, evaluated in DSS-induced mouse model, compared with hADSC/cADSC suspension; Body weight was measured. Seven days after administration, mice were sacrificed, colon length was measured, and histological evaluation was conducted. (B) Safety test: Tumorigenicity and toxicity tests were conducted. cADSC CellSaic evaluated in NOG mouse. Volume of nodules, body weight, and food consumption were measured until 8 weeks. Four and 8 weeks after administration, urinalysis, hematology test, body chemistry test, necropsy organ weight measurement, and histopathological examination were conducted.

#### Administration of CellSaics and suspension

At day 7 after administration of DSS water, the mice were randomized and injected with implants intraperitoneally using an 18G needle or intravenously under general isoflurane anesthesia. The Animal Care Committee of FUJIFILM Corporation approved the experimental protocol, and all experimental procedures used in animal studies were performed in accordance with international guidelines.

#### Groups

For the hADSC test, transplantation was performed using the same i.p. injection method to compare the efficacy of the hADSC CellSaic and hADSC suspension. The study groups were as follows.

1. Non-DSS control group (*n* = 5; no treatment with DSS)
2. DSS control group (*n* = 5; no cell transplantation; only 400 µL Bulletkit ADSC medium)
3. hADSC suspension i.p. group (*n* = 5; hADSC suspension [1.2 × 10^6^ cells in 400 µL Bulletkit ADSC medium])
4. hADSC CellSaic i.p. group (*n* = 5; hADSC CellSaics [total 1.2 × 10^6^ cells and 1 mg of RCP petaloid µ-pieces in 400 µL Bulletkit ADSC medium])

For the cADSC test, the efficacy of i.p. injection of cADSC CellSaics was compared with that of i.v. injection of cADSC suspension used at a clinical dose. Clinical dose cell counting was conducted as described previously^15^. To assess the efficacy of cADSCs by the cell counting method, i.v. administration of cADSCs, at the same cell count as cADSC CellSaics, was also conducted.

1. Non-DSS control group (*n* = 5; no treatment with DSS)
2. DSS control group (*n* = 5; no cell transplantation (only 400 µL cADSC medium)
3. cADSC suspension clinical dose i.v. group (*n* = 5; cADSC suspension [4 × 10^4^ cells in 400 µL cADSC medium])
4. cADSC suspension i.v. group (*n* = 4; cADSC suspension [1.2 × 10^6^ cells in 400 µL cADSC medium])
5. cADSC CellSaic i.p. group (*n* = 4; cADSC CellSaics [total 1.2 × 10^6^ cells and 0.25 mg of RCP petaloid µ-pieces in 400 µL cADSC medium])

### Outcome Measurements

#### Weight recovery

For evaluation, the body weights were recorded daily. The weight recovery percentage was calculated as (the body weight at the point) / (the initial body weight) × 100.

#### Colon length

Seven days after transplantation, the mice were sacrificed, and colon length measurements and histological evaluations were conducted. After the colon was removed, the colon length was measured from the caecum to the anus.

#### Histological evaluation of inflammatory score

To assess the inflammatory level, histological sections were prepared 7 days following implantation. Colon tissues were removed, cut into three parts (upper colon, lower colon, rectum), and fixed with 10% phosphate-buffered formalin. Hematoxylin and eosin (H&E)-stained sections were prepared for histological examination, with the center of the three parts facing the outside. The inflammatory scores of the colon sections were calculated using a scoring system. (A) Depth of ulceration, (B) area of ulceration, (C) edema, and (D) infiltration were scored individually for the samples using a graded scale of 0–4, as described in Table 1. The scores were reviewed by a pathologist and veterinarian at FUJIFILM Corp. The histological score was defined as the sum of the four parameters (A+B+C+D) of the three sections and represents the average of the effective values.

**Table 1.** Scoring System for Histological Evaluation.

A	Depth of ulceration
0	normal morphology of epithelium
1	partial destruction of mucosa
2	overall destruction of mucosa
3	destruction of muscularis mucosa layer
4	destruction of submucosa
B	Area of ulceration
0	no ulceration
1	ulceration area < 25%
2	25% ≤ ulceration area < 50%
3	50% ≤ ulceration area < 75%
4	75% ≤ ulceration area
C	Edema
0	no edema
1	slight
2	moderate
3	severe
4	more severe
D	Infiltration
0	within normal limit
1	slight
2	moderate
3	severe
4	more severe

### *In Vitro* Analysis

#### Cell culture level of TSG-6 analysis by ELISA

The TSG-6 analysis was performed as described previously^23^. hADSC CellSaic was prepared by mixing hADSCs (5 × 10^4^ cells/mL) and RCP pieces (0.05 mg/mL) in DMEM; this mixture was seeded on a PrimeSurface 96U plate (Sumitomo Bakelite Co. Ltd., Tokyo, Japan) in 200 μL wells. For spheroid formation, hADSCs in DMEM were prepared using 5 × 10^4^ cells/mL, and the cells were seeded on a PrimeSurface 96 U plate in 200 μL wells. Each plate was centrifuged using a tabletop plate centrifuge (600 ×*g*, 5 min) and then incubated. At day 1, the medium was exchanged with DMEM containing 10 ng/mL TNFα or DMEM without TNFα. After 48 h, the levels of TSG-6 in each supernatant were measured with a RayBio Human TSG-6 ELISA Kit (ELH-TSG6; RayBiotech, Norcross, GA, USA).

### Safety Test

#### Tumorigenicity and Toxicity Tests (Fig. 1B)

The test was conducted by Nihon Bioresearch Center, Inc. (Hashima, Japan).

#### Population

Male and female NOG mice at 6 weeks of age (CLEA Japan, Inc., Meguro-ku, Japan) were used in the test; 36 male mice and 36 female mice were used in this test.

#### Groups

Mice were grouped as follows. Note that male group and female group were respectively prepared for each of the three groups.

1. Control (saline) group (*n* = 12; 0.4 mL physiological saline])
2. Control (culture media) group (*n* = 12; 0.4 mL cADSC media])
3. CellSaic group (*n* = 12; cADSC CellSaics [total 1 × 10^7^ cells and 1.04 mg of µ-pieces in 0.4 mL cADSC media])

Physiological saline (Otsuka Pharmaceutical Co., Ltd., Tokyo, Japan), culture medium used for cADSCs, or CellSaics (10^7^ cells cADSC and 1.04 mg µ-pieces) was administered at a volume of 0.4 mL to the right lateral abdomen of mice under anesthesia using a 23G injection needle.

#### Outcome measurements

After administration, the sizes of subcutaneous nodules were measured over time using a digital caliper (CD-15, Mitsutoyo Corporation, Kasugai, Japan) until 56 days after administration. Volume of nodules was calculated using the following formula: (long diameter × short diameter^2^)/2. The means and standard deviations of subcutaneous volume of nodules were calculated for each group. Other details related to general toxicity study are described in the Supplemental Materials and Methods.

### Statistical Analyses

The results are presented as the means ± standard deviation. Colon length and histological score were analyzed using the *t* test. Body weight changes were analyzed using one-way analysis of variance with Dunnett’s test. Tumorigenicity was analyzed using Steel–Dwass test. The significance level was set at 5%, and the values were presented in three groups of less than 5% (*p < 0.05), less than 1% (**p < 0.01), and less than 0.5% (***p < 0.005).

## Results

### hADSC CellSaic Recovers Body Weight and Colon Length in DSS-Induced Mice

**Fig. 2.**
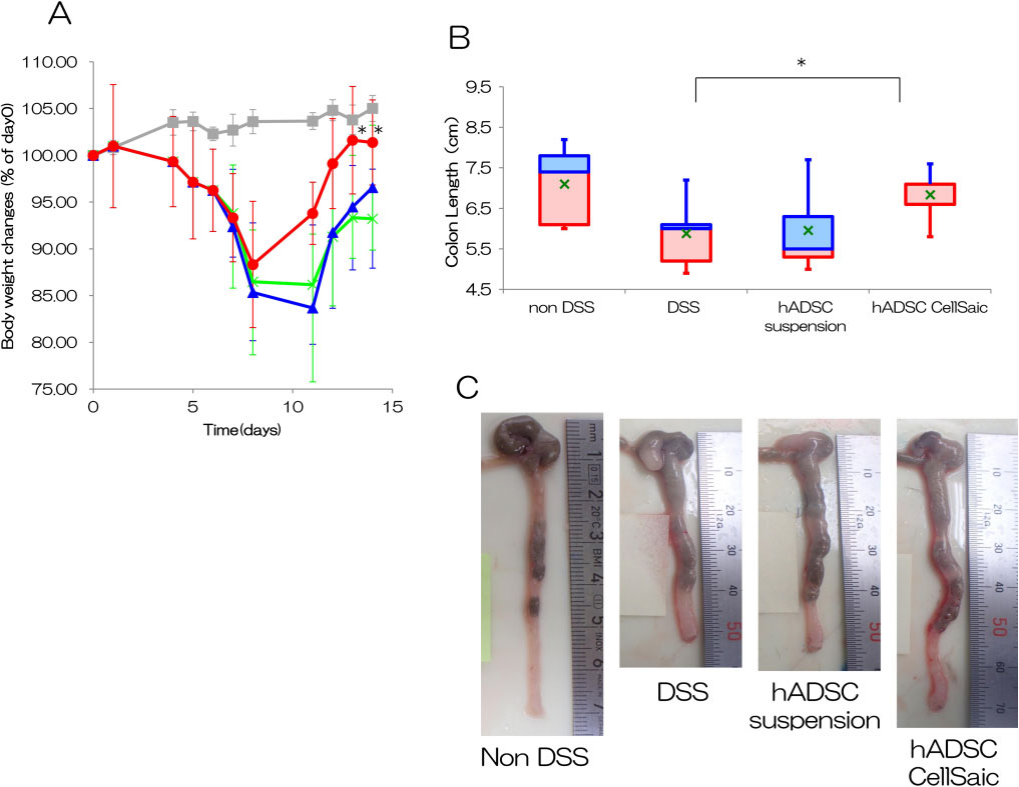
hADSC CellSaic attenuates recovery of body weight and colon length. (A) Percentage of body weight changes. Non-DSS group [no treatment with DSS, *n* = 5 (gray square)], DSS group [no cell transplantation into DSS mice, *n* = 5 (green cross)], hADSC suspension i.p. group [1.2 × 10^6^ hADSCs in suspension intraperitoneally administered to DSS mice, *n* = 5 (blue triangle)], hADSC CellSaic i.p. group [CellSaics of 1.2 × 10^6^ hADSCs and 1 mg u-pieces, intraperitoneally administered to DSS mice, *n* = 5 (red circle)]. *p < 0.05. The hADSC CellSaic i.p. group and hADSC suspension i.p. group were each compared with the DSS group by one-way analysis of variance with Dunnett’s test. (B) Colon length on day 14. The value of 75% – median (blue box), median – the value of 25% (red box), value of average (green cross). Non-DSS group [no treatment with DSS, *n* = 5], DSS group [no cell transplantation into DSS mice, *n* = 5], hADSC suspension i.p. group [1.2 × 10^6^ hADSCs in suspension intraperitoneally administered to DSS mice, *n* = 5], hADSC CellSaic i.p. group [CellSaics of 1.2 × 10^6^ hADSCs and 1 mg u-pieces, intraperitoneally administered to DSS mice, *n* = 5]. *p < 0.05. hADSC CellSaic group and hADSC suspension group compared with the DSS group. (C) The images of colon tissues. Representative images of colon tissue in each group were shown.

We examined the anti-inflammatory effects of hADSC CellSaic using a DSS-induced model of colitis. Oral administration of 3% DSS for 7 days induced acute colitis in C57BL/6 mice, which was confirmed by the reduction of body weight (Fig. 2A). In the DSS group that did not receive cell injection, body weight decreased to 86% at day 11 and recovered to only 93% at day 14. In the hADSC suspension i.p. group, following administration of hADSCs alone at day 7, body weight decreased to 84% at day 11 but recovered to 97% at day 14; this group showed little improvement in body weight recovery. In the hADSC CellSaic i.p. group, body weight increased from day 8 to 11, recovering to 94% at day 11 and 101% at day 14, reaching 105% of the non-DSS group. The hADSC CellSaic i.p. group showed significant differences in body weight on days 13 and 14 (*p* < 0.05). The hADSC suspension i.p. group showed no significant differences. The hADSC suspension i.p. group showed no significant differences. These results indicate that the CellSaic platform promoted body weight recovery. The mean colon lengths at day 14, 7 days after transplantation, were 5.8 cm in the DSS group and 6.0 cm in the hADSC suspension i.p. group; colon length was significantly longer at 6.8 cm in the hADSC CellSaic i.p. group (Fig. 2B). Colon length was 7.1 cm in the non-DSS group, which is close to the length in the healthy group. The images of colon tissues are shown in Fig. 2C. The hADSC CellSaic i.p. group showed significant differences in colon length from the DSS group (*p* < 0.05). The hADSC suspension i.p. group showed no significant differences. The recovery of body weight and colon length indicate that hADSC CellSaics were more effective than the hADSC suspension.

### hADSC CellSaic Decreases Ulceration and Inflammatory Cells

**Fig. 3.**
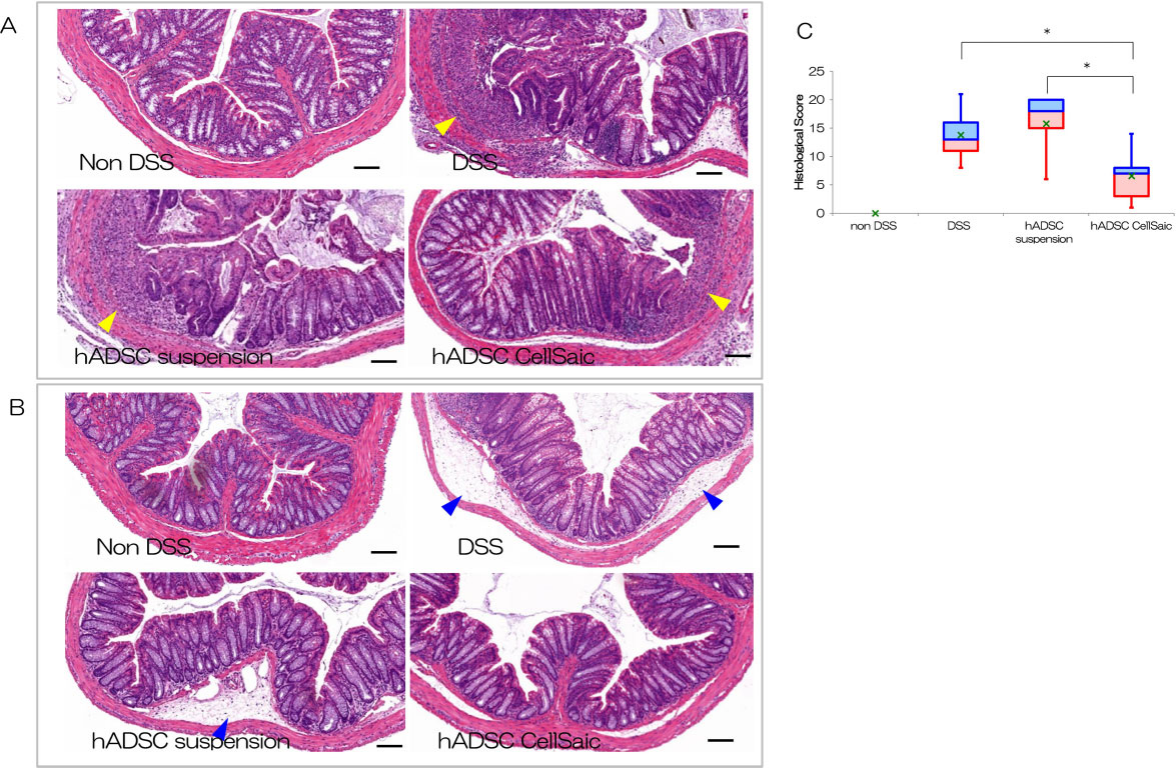
ADSC CellSaic decreased inflammatory cells and repaired ulceration in histological analysis. Histopathological comparison of colitis in lower colon at day 14. There was no ulceration and no infiltration in the non-DSS group. In the DSS group and hADSC suspension group, ulceration and infiltration are more severe than in the hADSC CellSaic group. The arrows indicate ulceration and infiltration. Scale Bar is 100 µm. (A) Histopathological comparison of colitis in the rectum at day 14. There was no edema in the non-DSS group. In the DSS group and hADSC suspension group, edema was more severe than in the hADSC CellSaic group. The arrows indicate edema. Scale bar is 100 µm. (B) Histological evaluation of the colon at day 14. The value of 75% – median (blue box), median – the value of 25% (red box), the value of average (green cross). Non-DSS group [no treatment with DSS, *n* = 5], DSS group [no cell transplantation into DSS mice, *n* = 5], hADSC suspension i.p. group [1.2 × 10^6^ hADSCs in suspension intraperitoneally administered to DSS mice, *n* = 5], hADSC CellSaic i.p. group [CellSaics of 1.2 × 10^6^ hADSCs and 1 mg u-pieces, intraperitoneally administered to DSS mice, *n* = 5]. *p < 0.05. hADSC CellSaic group compared with DSS group and hADSC suspension group.

We evaluated pathological sections to determine the depth and area of ulceration, edema, and infiltration of inflammatory cells. Pathological specimens are shown in Figs. 3A and 3B. There was no ulceration or infiltration in the non-DSS group. In the DSS group and the hADSC suspension group, ulceration and infiltration were more severe than in the hADSC CellSaic group. Scoring results are presented in Fig. 3C. Mean total scores were 0 in the non-DSS group, 13.8 in the DSS group, 15.8 in the hADSC suspension i.p. group, and 6.6 in the hADSC CellSaic i.p. group, confirming the narrower area of ulceration, smaller areas of edema, and reduced infiltration of inflammatory cells in the hADSC CellSaic i.p. group. Histological examination showed that hADSC CellSaic attenuates inflammation and disruption of the crypt architecture.

### cADSC CellSaic Promotes Improvement of DSS-Induced Colitis

**Fig. 4.**
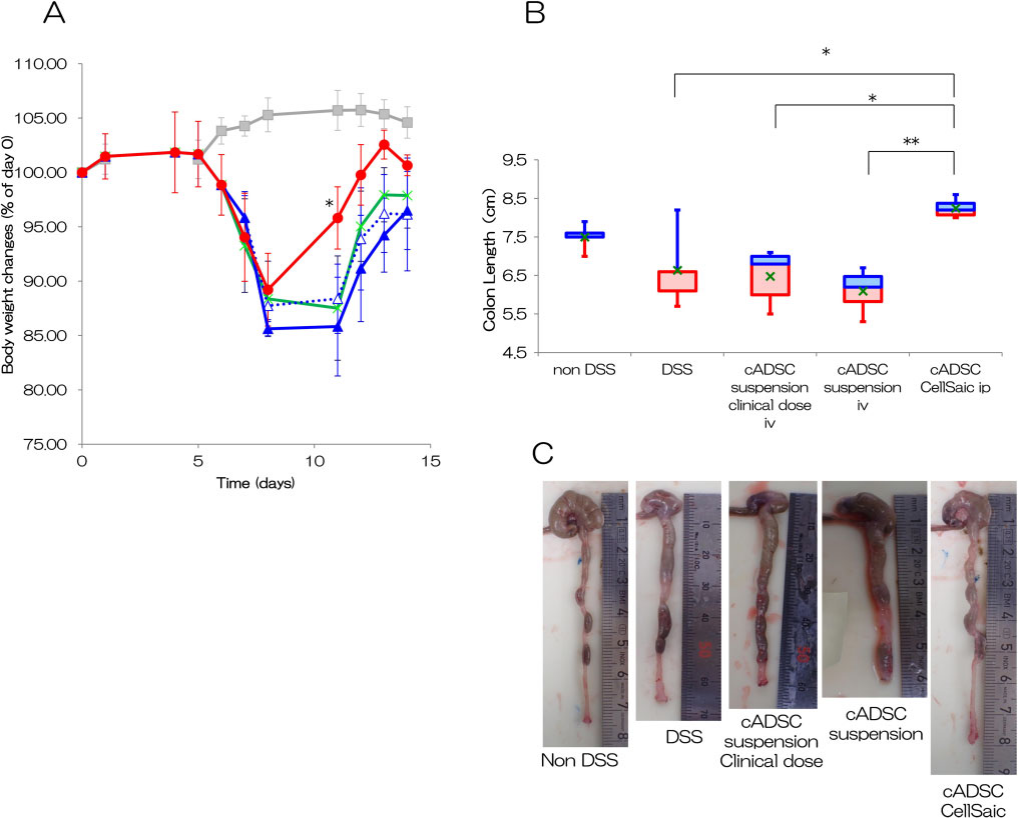
cADSC CellSaic also attenuates recovery of body weight and colon length. (A) Percentage of body weight changes. Non-DSS group [no treatment with DSS, *n* = 5 (gray square)], DSS group [no cell transplantation into DSS mice, *n* = 5 (green cross)], cADSC suspension i.v. group [1.2 × 10^6^ cADSCs in suspension intravenously administered to DSS mice, *n* = 4 (close blue triangle solid line)], cADSC in suspension clinical dose i.v. group [4 × 10^4^ cADSCs in suspension intravenously administered to DSS mice, *n* = 5 (open blue triangle dotted line)], cADSC CellSaic group [CellSaics of 1.2 × 10^6^ cADSC and 0.25 mg u-pieces, intraperitoneally administered to DSS mice, *n* = 4 (red circle)]. *p < 0.05. The cADSC CellSaic i.p. group, cADSC suspension i.v. group, and cADSC suspension clinical dose i.v. group were each compared with the DSS group by one-way analysis of variance with Dunnett’s test. (B) Colon length of day 14. The value of 75% – median (blue box), median – the value of 25% (red box), value of average (green cross). Non-DSS group [no treatment with DSS, *n* = 5], DSS group [no cell transplantation into DSS mice, *n* = 5], cADSC suspension i.v. group [1.2 × 10^6^ cADSCs in suspension intravenously administered to DSS mice, *n* = 4], cADSCs in suspension clinical dose i.v. group [4 × 10^4^ cADSCs in suspension intravenously administered to DSS mice, *n* = 5], cADSC CellSaic group [CellSaics of 1.2 × 10^6^ cells cADSCs and 0.25 mg u-pieces, intraperitoneally administered to DSS mice, *n* = 4]. *p < 0.05. **p < 0.01. cADSC CellSaic group compared with DSS group, cADSC suspension i.v. group, and cADSC suspension clinical dose i.v. group. (C) The images of colon tissues. Representative images of colon tissue in each group were shown.

A test similar to that used to assess the efficacy of hADSCs was performed using cADSCs. Our results indicate that the body weights in the DSS group, the cADSC suspension i.v. group, and the cADSC suspension clinical dose i.v. group were 86–88% at days 8 and 11, and 96–98% at day 14, respectively (Fig. 4A). In addition, the body weights in the cADSC CellSaic i.p. group were 89% at day 8, 96% at day 11, and 101% at day 14, indicating a recovery in body weight. The cADSC CellSaic i.p. group showed significant differences in body weight on day 11 compared with the DSS group (*p* < 0.05). The cADSC suspension i.v. group and the cADSC suspension clinical dose i.v. group showed no significant differences. Colon lengths were 6.7 cm in the DSS group, 6.1 cm in the cADSC suspension i.v. group, 6.5 cm in the cADSC suspension clinical dose i.v. group, and 8.3 cm in the cADSC CellSaic i.p. group, confirming significant colon length recovery in the cADSC CellSaic i.p. group (Fig. 4B). The images of colon tissues are shown in Fig. 4C. The cADSC CellSaic i.p. group showed significant differences in colon length compared with the DSS group (*p* < 0.05), the cADSC suspension clinical dose i.v. group (*p* < 0.05), and the cADSC suspension i.v. group (*p* < 0.01).

### hADSC CellSaic secretes more TSG-6 than does hADSC only *in vitro*

**Fig. 5.**
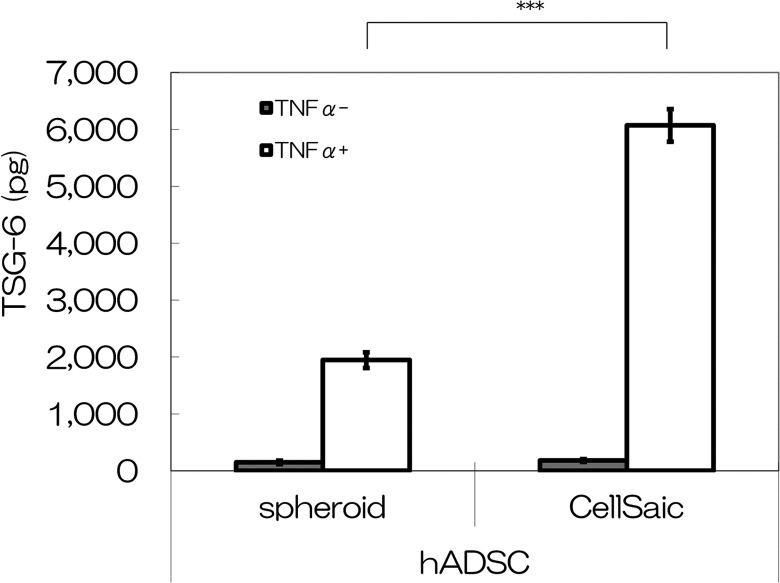
Comparison of TSG-6 levels *in vitro*. TSG-6 level in the supernatants collected from hADSC spheroids and hADSC CellSaic, after incubation with or without TNFα for 48 h, as measured by ELISA. ***p < 0.005.

Secretion of the anti-inflammatory protein TSG-6 was tested under the culture condition of hADSC CellSaic and hADSC spheroid. The TSG-6 secretion levels after 48 h of incubation were 6072 pg for the hADSC CellSaic and 1946 pg for the hADSC sphenoid in culture medium containing TNFα (Fig. 5). These results indicate that more TSG-6 was secreted in the presence of hADSC CellSaic. The hADSC CellSaic showed significant differences from the hADSC spheroid (*p* < 0.005).

### cADSC CellSaic has no Tumorigenicity

**Fig. 6.**
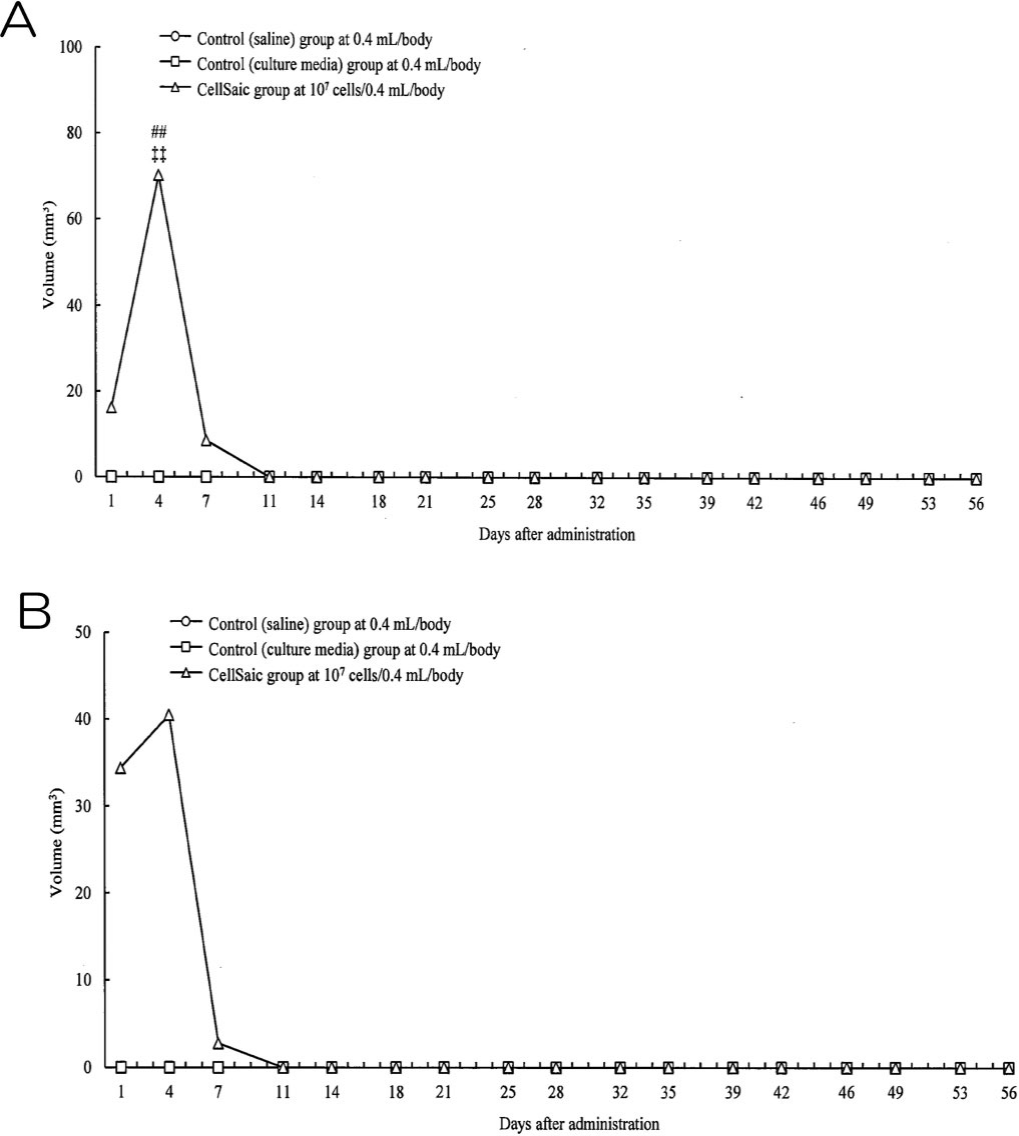
Volume of nodules in tumorigenicity test. (A) Volume of nodules in male mice. Control (saline) group; circle, Control (culture media) group: square, CellSaic group; triangle. CellSaic group was significantly different from the control (saline) group (##: *p* < 0.01 by Steel–Dwass test) and control (culture media) group (++: *p* < 0.01 by Steel–Dwass test). (B) Volume of nodules in female mice. Control (saline) group; circle, Control (culture media) group: square, CellSaic group; triangle.

The safety of subcutaneous administration was assessed by conducting a tumorigenicity test in NOG mice. Nodules were observed in female and male mice in the CellSaic group at days 1, 4, and 7, reaching maximum volumes of 70.21 mm^2^ in male mice and 40.45 mm^2^ in female mice at day 4. However, similar to the physiological saline control group and the culture medium control group, subcutaneous nodules were not visible in the male and female mice of the cADSC CellSaic group at day 11 (Figs. 6A and 6B). Histological evaluation of the administration sites at 4 and 8 weeks revealed no cell division despite the presence of a remnant at the administration site; this indicates that no tumor development occurred in this group. In addition, no significant intergroup differences were observed in biochemistry testing, hematological testing, organ weight, food consumption, or body weight. No abnormalities were found during pathological evaluation of the organs in the cADSC CellSaic group at 4 and 8 weeks (Supplemental results; all Figures S1–S4 and Tables S4–S29).

## Discussion

Our mouse models of DSS-induced colitis demonstrated that human and canine ADSC CellSaic exerted higher therapeutic effects than cells injected alone. A previous study reported the effect of CellSaic^21^. The presence of a scaffold for cells can prevent anoikis, which is apoptosis of adherent cells induced by the absence of a scaffold.

With respect to preventing anoikis, we considered that hADSC CellSaic showed good survival intraperitoneally compared with hADSCs in suspension delivered as single cells due to adhesion to the µ-pieces in hADSC CellSaic. Indeed, we intraperitoneally injected the hADSC CellSaics with labeled cells and confirmed their behavior *in vivo* using an *in vivo* imaging system. We confirmed that hADSCs, delivered via CellSaics by i.p. administration, survived for 28 days or more (Supplemental results; FigureS5). In contrast, most cells administered alone disappear in a few days^23^. Furthermore, the hADSC CellSaics likely released more cytokines than did hADSCs only.

In our *in vitro* experiment, TSG-6 by hADSC CellSaic secretion was 3.1-fold greater than that of hADSCs only. In pathological evaluation, edema and infiltration of inflammatory cells in the intestine were inhibited in the hADSC CellSaic group. The abundant secretion of TSG-6 from the hADSC CellSaics may have resulted in enhanced improvement. TSG-6 is an important anti-inflammatory cytokine that exerts its effects in various models of inflammation. TSG-6 is a component of a negative feedback mechanism capable of downregulating the inflammatory response^24^. Moreover, TSG-6 is sufficient to reduce intestinal inflammation in mice with colitis. TSG-6 may play a major role in the anti-inflammatory mechanism used by BMSCs. In the i.p. administration of BMSCs group, which showed highest efficacy of all groups, the serum level of TSG-6 was the highest among the mouse models of DSS-induced colitis^16^. Thus the therapeutic effects of ADSC CellSaic may be mainly anti-inflammatory effects.

Angiogenesis contributes to repair the tissue. During chronic inflammatory diseases, inhibition of angiogenesis attenuates further inflammation and disease pathology^25^. Angiogenesis plays an important role as a protective factor during the regeneration of injured tissues. Intraperitoneal administration of recombinant human hepatocyte growth factor, HGF, which has been shown to be a potent angiogenic factor, can accelerate colonic mucosal repair in rats with DSS-induced colitis^26^. In pathological evaluation, repair of ulcer and epithelial tissue structure was promoted more in the hADSC CellSaic group than in the DSS control group. In addition, BMSC CellSaic promotes angiogenesis as compared with using BMSCs alone^21^. Thus, the promotion of angiogenesis may have contributed to the repair of intestinal tissue, although firm evidence was not obtained in this study. The cADSC CellSaics had the same effect as hADSC CellSaics. In contrast, i.v. administration of the cADSC suspension showed no effect either at the clinical dose or same cell count as that used with the CellSaic. This observation would be caused by the reason mentioned in hADSC above.

Tumorigenicity analysis using NOG mice administered cADSC CellSaics showed no formation of subcutaneous nodules and cell division of administered cells after 4 and 8 weeks, suggesting that there is no serious risk of tumor development. Moreover, there were no abnormal findings including tumor development in systemic organs, indicating no serious risk of toxicity. In summary, canine ADSC CellSaics may be safe for veterinary therapies.

We found that human and canine ADSCs, administered as CellSaic, are effective for DSS colitis therapy. In addition, we confirmed no tumorigenicity or toxicity for cADSC CellSaic. Based on these findings, these results may be clinically applied for colitis therapy of dogs; moreover, clinical studies of companion dogs will accelerate the clinical development of therapies for humans.

## Supplementary Material

Supplementary material

Supplementary material

Supplementary material
