# Supplementary material for: The Therapeutic Effects of Adipose-Derived Stem Cells and Recombinant Peptide Pieces on Mouse Model of DSS Colitis
Source: Cell Transplant. 2018 Jul 6;27(9):1390–400. doi: 10.1177/0963689718782442 (PMC6168991; doi:10.1177/0963689718782442)
Supplement: Supplementary material [file 782442_Supplemental_material_2.docx]

Table S4. Clinical parameters in male mice.

|  |  |  |  |  |  |  |  |  |  |  |  |  |  |  |  |  |  |  |  |  |  |  |  |  |  |  |  |  |  |  |  |  |  |  |  |  |  |  |  |  |  |  |  |  |
| --- | --- | --- | --- | --- | --- | --- | --- | --- | --- | --- | --- | --- | --- | --- | --- | --- | --- | --- | --- | --- | --- | --- | --- | --- | --- | --- | --- | --- | --- | --- | --- | --- | --- | --- | --- | --- | --- | --- | --- | --- | --- | --- | --- | --- |
| Group | Dose | Number of animals | Pre |  | Hours | | | | |  | Days after administration | | | | | | | | | | | | | | | | | | | | | | | | | | | | | | | | |  |
|  |  | and clinical signs |  |  | 0-1 |  | 1-2 |  | 2-4 |  | 1 |  | 2 |  | 3 |  | 4 |  | 5 |  | 6 |  | 7 |  | 8 |  | 9 |  | 10 |  | 11 |  | 12 |  | 13 |  | 14 |  | 15 |  | 16 |  | 17 |  |
| Control | 0.4 mL/body | Number of animals | 12 |  | 12 |  | 12 |  | 12 |  | 12 |  | 12 |  | 12 |  | 12 |  | 12 |  | 12 |  | 12 |  | 12 |  | 12 |  | 12 |  | 12 |  | 12 |  | 12 |  | 12 |  | 12 |  | 12 |  | 12 |  |
| (saline) |  | Normal | 12 |  | 0 |  | 0 |  | 12 |  | 12 |  | 12 |  | 12 |  | 12 |  | 12 |  | 12 |  | 12 |  | 12 |  | 12 |  | 12 |  | 12 |  | 12 |  | 12 |  | 12 |  | 12 |  | 12 |  | 12 |  |
|  |  | Nodule, injection site | 0 |  | 12 |  | 12 |  | 0 |  | 0 |  | 0 |  | 0 |  | 0 |  | 0 |  | 0 |  | 0 |  | 0 |  | 0 |  | 0 |  | 0 |  | 0 |  | 0 |  | 0 |  | 0 |  | 0 |  | 0 |  |
| Control | 0.4 mL/body | Number of animals | 12 |  | 12 |  | 12 |  | 12 |  | 12 |  | 12 |  | 12 |  | 12 |  | 12 |  | 12 |  | 12 |  | 12 |  | 12 |  | 12 |  | 12 |  | 12 |  | 12 |  | 12 |  | 12 |  | 12 |  | 12 |  |
| (culture media) |  | Normal | 12 |  | 0 |  | 0 |  | 12 |  | 12 |  | 12 |  | 12 |  | 12 |  | 12 |  | 12 |  | 12 |  | 12 |  | 12 |  | 12 |  | 12 |  | 12 |  | 12 |  | 12 |  | 12 |  | 12 |  | 12 |  |
|  |  | Nodule, injection site | 0 |  | 12 |  | 12 |  | 0 |  | 0 |  | 0 |  | 0 |  | 0 |  | 0 |  | 0 |  | 0 |  | 0 |  | 0 |  | 0 |  | 0 |  | 0 |  | 0 |  | 0 |  | 0 |  | 0 |  | 0 |  |
| CellSaic | 10^7^ cells/0.4 mL/body | Number of animals | 12 |  | 12 |  | 12 |  | 12 |  | 12 |  | 12 |  | 12 |  | 12 |  | 12 |  | 12 |  | 12 |  | 12 |  | 12 |  | 12 |  | 12 |  | 12 |  | 12 |  | 12 |  | 12 |  | 12 |  | 12 |  |
|  |  | Normal | 12 |  | 0 |  | 0 |  | 12 |  | 9 |  | 9 |  | 5 |  | 4 |  | 4 |  | 4 |  | 9 |  | 9 |  | 9 |  | 9 |  | 12 |  | 12 |  | 12 |  | 12 |  | 12 |  | 12 |  | 12 |  |
|  |  | Nodule, injection site | 0 |  | 12 |  | 12 |  | 0 |  | 3 |  | 3 |  | 7 |  | 8 |  | 8 |  | 8 |  | 3 |  | 3 |  | 3 |  | 3 |  | 0 |  | 0 |  | 0 |  | 0 |  | 0 |  | 0 |  | 0 |  |
| Pre: Before administration. | |  |  |  |  |  |  |  |  |  |  |  |  |  |  |  |  |  |  |  |  |  |  |  |  |  |  |  |  |  |  |  |  |  |  |  |  |  |  |  |  |  |  |  |
| Hours: Hours after administration | |  |  |  |  |  |  |  |  |  |  |  |  |  |  |  |  |  |  |  |  |  |  |  |  |  |  |  |  |  |  |  |  |  |  |  |  |  |  |  |  |  |  |  |
|  |  |  |  |  |  |  |  |  |  |  |  |  |  |  |  |  |  |  |  |  |  |  |  |  |  |  |  |  |  |  |  |  |  |  |  |  |  |  |  |  |  |  |  |  |
| Group | Dose | Number of animals | Days after administration | | | | | | | | | | | | | | | | | | | | | | | | | | | | | | | | | | | | | | | | |  |
|  |  | and clinical signs | 18 |  | 19 |  | 20 |  | 21 |  | 22 |  | 23 |  | 24 |  | 25 |  | 26 |  | 27 |  | 28 |  | 29 |  | 30 |  | 31 |  | 32 |  | 33 |  | 34 |  | 35 |  | 36 |  | 37 |  | 38 |  |
| Control | 0.4 mL/body | Number of animals | 12 |  | 12 |  | 12 |  | 12 |  | 12 |  | 12 |  | 12 |  | 12 |  | 12 |  | 12 |  | 12 |  | 6 |  | 6 |  | 6 |  | 6 |  | 6 |  | 6 |  | 6 |  | 6 |  | 6 |  | 6 |  |
| (saline) |  | Normal | 12 |  | 12 |  | 12 |  | 12 |  | 12 |  | 12 |  | 12 |  | 12 |  | 12 |  | 12 |  | 12 |  | 6 |  | 6 |  | 6 |  | 6 |  | 6 |  | 6 |  | 6 |  | 6 |  | 6 |  | 6 |  |
|  |  | Nodule, injection site | 0 |  | 0 |  | 0 |  | 0 |  | 0 |  | 0 |  | 0 |  | 0 |  | 0 |  | 0 |  | 0 |  | 0 |  | 0 |  | 0 |  | 0 |  | 0 |  | 0 |  | 0 |  | 0 |  | 0 |  | 0 |  |
| Control | 0.4 mL/body | Number of animals | 12 |  | 12 |  | 12 |  | 12 |  | 12 |  | 12 |  | 12 |  | 12 |  | 12 |  | 12 |  | 12 |  | 6 |  | 6 |  | 6 |  | 6 |  | 6 |  | 6 |  | 6 |  | 6 |  | 6 |  | 6 |  |
| (culture media) |  | Normal | 12 |  | 12 |  | 12 |  | 12 |  | 12 |  | 12 |  | 12 |  | 12 |  | 12 |  | 12 |  | 12 |  | 6 |  | 6 |  | 6 |  | 6 |  | 6 |  | 6 |  | 6 |  | 6 |  | 6 |  | 6 |  |
|  |  | Nodule, injection site | 0 |  | 0 |  | 0 |  | 0 |  | 0 |  | 0 |  | 0 |  | 0 |  | 0 |  | 0 |  | 0 |  | 0 |  | 0 |  | 0 |  | 0 |  | 0 |  | 0 |  | 0 |  | 0 |  | 0 |  | 0 |  |
| CellSaic | 10^7^ cells/0.4 mL/body | Number of animals | 12 |  | 12 |  | 12 |  | 12 |  | 12 |  | 12 |  | 12 |  | 12 |  | 12 |  | 12 |  | 12 |  | 6 |  | 6 |  | 6 |  | 6 |  | 6 |  | 6 |  | 6 |  | 6 |  | 6 |  | 6 |  |
|  |  | Normal | 12 |  | 12 |  | 12 |  | 12 |  | 12 |  | 12 |  | 12 |  | 12 |  | 12 |  | 12 |  | 12 |  | 6 |  | 6 |  | 6 |  | 6 |  | 6 |  | 6 |  | 6 |  | 6 |  | 6 |  | 6 |  |
|  |  | Nodule, injection site | 0 |  | 0 |  | 0 |  | 0 |  | 0 |  | 0 |  | 0 |  | 0 |  | 0 |  | 0 |  | 0 |  | 0 |  | 0 |  | 0 |  | 0 |  | 0 |  | 0 |  | 0 |  | 0 |  | 0 |  | 0 |  |
|  |  |  |  |  |  |  |  |  |  |  |  |  |  |  |  |  |  |  |  |  |  |  |  |  |  |  |  |  |  |  |  |  |  |  |  |  |  |  |  |  |  |  |  |  |
|  |  |  |  |  |  |  |  |  |  |  |  |  |  |  |  |  |  |  |  |  |  |  |  |  |  |  |  |  |  |  |  |  |  |  |  |  |  |  |  |  |  |  |  |  |
|  |  |  |  |  |  |  |  |  |  |  |  |  |  |  |  |  |  |  |  |  |  |  |  |  |  |  |  |  |  |  |  |  |  |  |  |  |  |  |  |  |  |  |  |  |
| Group | Dose | Number of animals | Days after administration | | | | | | | | | | | | | | | | | | | | | | | | | | | | | | | | | | |  |  |  |  |  |  |  |
|  |  | and clinical signs | 39 |  | 40 |  | 41 |  | 42 |  | 43 |  | 44 |  | 45 |  | 46 |  | 47 |  | 48 |  | 49 |  | 50 |  | 51 |  | 52 |  | 53 |  | 54 |  | 55 |  | 56 |  |  |  |  |  |  |  |
| Control | 0.4 mL/body | Number of animals | 6 |  | 6 |  | 6 |  | 6 |  | 6 |  | 6 |  | 6 |  | 6 |  | 6 |  | 6 |  | 6 |  | 6 |  | 6 |  | 6 |  | 6 |  | 6 |  | 6 |  | 6 |  |  |  |  |  |  |  |
| (saline) |  | Normal | 6 |  | 6 |  | 6 |  | 6 |  | 6 |  | 6 |  | 6 |  | 6 |  | 6 |  | 6 |  | 6 |  | 6 |  | 6 |  | 6 |  | 6 |  | 6 |  | 6 |  | 6 |  |  |  |  |  |  |  |
|  |  | Nodule, injection site | 0 |  | 0 |  | 0 |  | 0 |  | 0 |  | 0 |  | 0 |  | 0 |  | 0 |  | 0 |  | 0 |  | 0 |  | 0 |  | 0 |  | 0 |  | 0 |  | 0 |  | 0 |  |  |  |  |  |  |  |
| Control | 0.4 mL/body | Number of animals | 6 |  | 6 |  | 6 |  | 6 |  | 6 |  | 6 |  | 6 |  | 6 |  | 6 |  | 6 |  | 6 |  | 6 |  | 6 |  | 6 |  | 6 |  | 6 |  | 6 |  | 6 |  |  |  |  |  |  |  |
| (culture media) |  | Normal | 6 |  | 6 |  | 6 |  | 6 |  | 6 |  | 6 |  | 6 |  | 6 |  | 6 |  | 6 |  | 6 |  | 6 |  | 6 |  | 6 |  | 6 |  | 6 |  | 6 |  | 6 |  |  |  |  |  |  |  |
|  |  | Nodule, injection site | 0 |  | 0 |  | 0 |  | 0 |  | 0 |  | 0 |  | 0 |  | 0 |  | 0 |  | 0 |  | 0 |  | 0 |  | 0 |  | 0 |  | 0 |  | 0 |  | 0 |  | 0 |  |  |  |  |  |  |  |
| CellSaic | 10^7^ cells/0.4 mL/body | Number of animals | 6 |  | 6 |  | 6 |  | 6 |  | 6 |  | 6 |  | 6 |  | 6 |  | 6 |  | 6 |  | 6 |  | 6 |  | 6 |  | 6 |  | 6 |  | 6 |  | 6 |  | 6 |  |  |  |  |  |  |  |
|  |  | Normal | 6 |  | 6 |  | 6 |  | 6 |  | 6 |  | 6 |  | 6 |  | 6 |  | 6 |  | 6 |  | 6 |  | 6 |  | 6 |  | 6 |  | 6 |  | 6 |  | 6 |  | 6 |  |  |  |  |  |  |  |
|  |  | Nodule, injection site | 0 |  | 0 |  | 0 |  | 0 |  | 0 |  | 0 |  | 0 |  | 0 |  | 0 |  | 0 |  | 0 |  | 0 |  | 0 |  | 0 |  | 0 |  | 0 |  | 0 |  | 0 |  |  |  |  |  |  |  |
|  |  |  |  |  |  |  |  |  |  |  |  |  |  |  |  |  |  |  |  |  |  |  |  |  |  |  |  |  |  |  |  |  |  |  |  |  |  |  |  |  |  |  |  |  |

Table S5. Clinical parameters in female mice

|  |  |  |  |  |  |  |  |  |  |  |  |  |  |  |  |  |  |  |  |  |  |  |  |  |  |  |  |  |  |  |  |  |  |  |  |  |  |  |  |  |  |  |  |  |
| --- | --- | --- | --- | --- | --- | --- | --- | --- | --- | --- | --- | --- | --- | --- | --- | --- | --- | --- | --- | --- | --- | --- | --- | --- | --- | --- | --- | --- | --- | --- | --- | --- | --- | --- | --- | --- | --- | --- | --- | --- | --- | --- | --- | --- |
| Group | Dose | Number of animals | Pre |  | Hours | | | | |  | Days after administration | | | | | | | | | | | | | | | | | | | | | | | | | | | | | | | | |  |
|  |  | and clinical signs |  |  | 0-1 |  | 1-2 |  | 2-4 |  | 1 |  | 2 |  | 3 |  | 4 |  | 5 |  | 6 |  | 7 |  | 8 |  | 9 |  | 10 |  | 11 |  | 12 |  | 13 |  | 14 |  | 15 |  | 16 |  | 17 |  |
| Control | 0.4 mL/body | Number of animals | 12 |  | 12 |  | 12 |  | 12 |  | 12 |  | 12 |  | 12 |  | 12 |  | 12 |  | 12 |  | 12 |  | 12 |  | 12 |  | 12 |  | 12 |  | 12 |  | 12 |  | 12 |  | 12 |  | 12 |  | 12 |  |
| (saline) |  | Normal | 12 |  | 0 |  | 0 |  | 12 |  | 12 |  | 12 |  | 12 |  | 12 |  | 12 |  | 12 |  | 12 |  | 12 |  | 12 |  | 12 |  | 12 |  | 12 |  | 12 |  | 12 |  | 12 |  | 12 |  | 12 |  |
|  |  | Nodule, injection site | 0 |  | 12 |  | 12 |  | 0 |  | 0 |  | 0 |  | 0 |  | 0 |  | 0 |  | 0 |  | 0 |  | 0 |  | 0 |  | 0 |  | 0 |  | 0 |  | 0 |  | 0 |  | 0 |  | 0 |  | 0 |  |
| Control | 0.4 mL/body | Number of animals | 12 |  | 12 |  | 12 |  | 12 |  | 12 |  | 12 |  | 12 |  | 12 |  | 12 |  | 12 |  | 12 |  | 12 |  | 12 |  | 12 |  | 12 |  | 12 |  | 12 |  | 12 |  | 12 |  | 12 |  | 12 |  |
| (culture media) |  | Normal | 12 |  | 0 |  | 0 |  | 12 |  | 12 |  | 12 |  | 12 |  | 12 |  | 12 |  | 12 |  | 12 |  | 12 |  | 12 |  | 12 |  | 12 |  | 12 |  | 12 |  | 12 |  | 12 |  | 12 |  | 12 |  |
|  |  | Nodule, injection site | 0 |  | 12 |  | 12 |  | 0 |  | 0 |  | 0 |  | 0 |  | 0 |  | 0 |  | 0 |  | 0 |  | 0 |  | 0 |  | 0 |  | 0 |  | 0 |  | 0 |  | 0 |  | 0 |  | 0 |  | 0 |  |
| CellSaic | 10^7^ cells/0.4 mL/body | Number of animals | 12 |  | 12 |  | 12 |  | 12 |  | 12 |  | 12 |  | 12 |  | 12 |  | 12 |  | 12 |  | 12 |  | 12 |  | 12 |  | 12 |  | 12 |  | 12 |  | 12 |  | 12 |  | 12 |  | 12 |  | 12 |  |
|  |  | Normal | 12 |  | 0 |  | 0 |  | 12 |  | 9 |  | 9 |  | 9 |  | 8 |  | 8 |  | 8 |  | 11 |  | 11 |  | 11 |  | 11 |  | 12 |  | 12 |  | 12 |  | 12 |  | 12 |  | 12 |  | 12 |  |
|  |  | Nodule, injection site | 0 |  | 12 |  | 12 |  | 0 |  | 3 |  | 3 |  | 3 |  | 4 |  | 4 |  | 4 |  | 1 |  | 1 |  | 1 |  | 1 |  | 0 |  | 0 |  | 0 |  | 0 |  | 0 |  | 0 |  | 0 |  |
| Pre: Before administration. | |  |  |  |  |  |  |  |  |  |  |  |  |  |  |  |  |  |  |  |  |  |  |  |  |  |  |  |  |  |  |  |  |  |  |  |  |  |  |  |  |  |  |  |
| Hours: Hours after administration | |  |  |  |  |  |  |  |  |  |  |  |  |  |  |  |  |  |  |  |  |  |  |  |  |  |  |  |  |  |  |  |  |  |  |  |  |  |  |  |  |  |  |  |
|  |  |  |  |  |  |  |  |  |  |  |  |  |  |  |  |  |  |  |  |  |  |  |  |  |  |  |  |  |  |  |  |  |  |  |  |  |  |  |  |  |  |  |  |  |
| Group | Dose | Number of animals | Days after administration | | | | | | | | | | | | | | | | | | | | | | | | | | | | | | | | | | | | | | | | |  |
|  |  | and clinical signs | 18 |  | 19 |  | 20 |  | 21 |  | 22 |  | 23 |  | 24 |  | 25 |  | 26 |  | 27 |  | 28 |  | 29 |  | 30 |  | 31 |  | 32 |  | 33 |  | 34 |  | 35 |  | 36 |  | 37 |  | 38 |  |
| Control | 0.4 mL/body | Number of animals | 12 |  | 12 |  | 12 |  | 12 |  | 12 |  | 12 |  | 12 |  | 12 |  | 12 |  | 12 |  | 12 |  | 6 |  | 6 |  | 6 |  | 6 |  | 6 |  | 6 |  | 6 |  | 6 |  | 6 |  | 6 |  |
| (saline) |  | Normal | 12 |  | 12 |  | 12 |  | 12 |  | 12 |  | 12 |  | 12 |  | 12 |  | 12 |  | 12 |  | 12 |  | 6 |  | 6 |  | 6 |  | 6 |  | 6 |  | 6 |  | 6 |  | 6 |  | 6 |  | 6 |  |
|  |  | Nodule, injection site | 0 |  | 0 |  | 0 |  | 0 |  | 0 |  | 0 |  | 0 |  | 0 |  | 0 |  | 0 |  | 0 |  | 0 |  | 0 |  | 0 |  | 0 |  | 0 |  | 0 |  | 0 |  | 0 |  | 0 |  | 0 |  |
| Control | 0.4 mL/body | Number of animals | 12 |  | 12 |  | 12 |  | 12 |  | 12 |  | 12 |  | 12 |  | 12 |  | 12 |  | 12 |  | 12 |  | 6 |  | 6 |  | 6 |  | 6 |  | 6 |  | 6 |  | 6 |  | 6 |  | 6 |  | 6 |  |
| (culture media) |  | Normal | 12 |  | 12 |  | 12 |  | 12 |  | 12 |  | 12 |  | 12 |  | 12 |  | 12 |  | 12 |  | 12 |  | 6 |  | 6 |  | 6 |  | 6 |  | 6 |  | 6 |  | 6 |  | 6 |  | 6 |  | 6 |  |
|  |  | Nodule, injection site | 0 |  | 0 |  | 0 |  | 0 |  | 0 |  | 0 |  | 0 |  | 0 |  | 0 |  | 0 |  | 0 |  | 0 |  | 0 |  | 0 |  | 0 |  | 0 |  | 0 |  | 0 |  | 0 |  | 0 |  | 0 |  |
| CellSaic | 10^7^ cells/0.4 mL/body | Number of animals | 12 |  | 12 |  | 12 |  | 12 |  | 12 |  | 12 |  | 12 |  | 12 |  | 12 |  | 12 |  | 12 |  | 6 |  | 6 |  | 6 |  | 6 |  | 6 |  | 6 |  | 6 |  | 6 |  | 6 |  | 6 |  |
|  |  | Normal | 12 |  | 12 |  | 12 |  | 12 |  | 12 |  | 12 |  | 12 |  | 12 |  | 12 |  | 12 |  | 12 |  | 6 |  | 6 |  | 6 |  | 6 |  | 6 |  | 6 |  | 6 |  | 6 |  | 6 |  | 6 |  |
|  |  | Nodule, injection site | 0 |  | 0 |  | 0 |  | 0 |  | 0 |  | 0 |  | 0 |  | 0 |  | 0 |  | 0 |  | 0 |  | 0 |  | 0 |  | 0 |  | 0 |  | 0 |  | 0 |  | 0 |  | 0 |  | 0 |  | 0 |  |
|  |  |  |  |  |  |  |  |  |  |  |  |  |  |  |  |  |  |  |  |  |  |  |  |  |  |  |  |  |  |  |  |  |  |  |  |  |  |  |  |  |  |  |  |  |
|  |  |  |  |  |  |  |  |  |  |  |  |  |  |  |  |  |  |  |  |  |  |  |  |  |  |  |  |  |  |  |  |  |  |  |  |  |  |  |  |  |  |  |  |  |
|  |  |  |  |  |  |  |  |  |  |  |  |  |  |  |  |  |  |  |  |  |  |  |  |  |  |  |  |  |  |  |  |  |  |  |  |  |  |  |  |  |  |  |  |  |
| Group | Dose | Number of animals | Days after administration | | | | | | | | | | | | | | | | | | | | | | | | | | | | | | | | | | |  |  |  |  |  |  |  |
|  |  | and clinical signs | 39 |  | 40 |  | 41 |  | 42 |  | 43 |  | 44 |  | 45 |  | 46 |  | 47 |  | 48 |  | 49 |  | 50 |  | 51 |  | 52 |  | 53 |  | 54 |  | 55 |  | 56 |  |  |  |  |  |  |  |
| Control | 0.4 mL/body | Number of animals | 6 |  | 6 |  | 6 |  | 6 |  | 6 |  | 6 |  | 6 |  | 6 |  | 6 |  | 6 |  | 6 |  | 6 |  | 6 |  | 6 |  | 6 |  | 6 |  | 6 |  | 6 |  |  |  |  |  |  |  |
| (saline) |  | Normal | 6 |  | 6 |  | 6 |  | 6 |  | 6 |  | 6 |  | 6 |  | 6 |  | 6 |  | 6 |  | 6 |  | 6 |  | 6 |  | 6 |  | 6 |  | 6 |  | 6 |  | 6 |  |  |  |  |  |  |  |
|  |  | Nodule, injection site | 0 |  | 0 |  | 0 |  | 0 |  | 0 |  | 0 |  | 0 |  | 0 |  | 0 |  | 0 |  | 0 |  | 0 |  | 0 |  | 0 |  | 0 |  | 0 |  | 0 |  | 0 |  |  |  |  |  |  |  |
| Control | 0.4 mL/body | Number of animals | 6 |  | 6 |  | 6 |  | 6 |  | 6 |  | 6 |  | 6 |  | 6 |  | 6 |  | 6 |  | 6 |  | 6 |  | 6 |  | 6 |  | 6 |  | 6 |  | 6 |  | 6 |  |  |  |  |  |  |  |
| (culture media) |  | Normal | 6 |  | 6 |  | 6 |  | 6 |  | 6 |  | 6 |  | 6 |  | 6 |  | 6 |  | 6 |  | 6 |  | 6 |  | 6 |  | 6 |  | 6 |  | 6 |  | 6 |  | 6 |  |  |  |  |  |  |  |
|  |  | Nodule, injection site | 0 |  | 0 |  | 0 |  | 0 |  | 0 |  | 0 |  | 0 |  | 0 |  | 0 |  | 0 |  | 0 |  | 0 |  | 0 |  | 0 |  | 0 |  | 0 |  | 0 |  | 0 |  |  |  |  |  |  |  |
| CellSaic | 10^7^ cells/0.4 mL/body | Number of animals | 6 |  | 6 |  | 6 |  | 6 |  | 6 |  | 6 |  | 6 |  | 6 |  | 6 |  | 6 |  | 6 |  | 6 |  | 6 |  | 6 |  | 6 |  | 6 |  | 6 |  | 6 |  |  |  |  |  |  |  |
|  |  | Normal | 6 |  | 6 |  | 6 |  | 6 |  | 6 |  | 6 |  | 6 |  | 6 |  | 6 |  | 6 |  | 6 |  | 6 |  | 6 |  | 6 |  | 6 |  | 6 |  | 6 |  | 6 |  |  |  |  |  |  |  |
|  |  | Nodule, injection site | 0 |  | 0 |  | 0 |  | 0 |  | 0 |  | 0 |  | 0 |  | 0 |  | 0 |  | 0 |  | 0 |  | 0 |  | 0 |  | 0 |  | 0 |  | 0 |  | 0 |  | 0 |  |  |  |  |  |  |  |
|  |  |  |  |  |  |  |  |  |  |  |  |  |  |  |  |  |  |  |  |  |  |  |  |  |  |  |  |  |  |  |  |  |  |  |  |  |  |  |  |  |  |  |  |  |

Table S6. Urinalysis in male mice (27 or 28 days after administration)

|  |  |  |  |  |  |  |  |  |  |  |  |  |  |  |  |  |  |
| --- | --- | --- | --- | --- | --- | --- | --- | --- | --- | --- | --- | --- | --- | --- | --- | --- | --- |
| Group |  |  | Control (saline) | | | | | Control (culture media) | | | | | CellSaic | | | | |
| Dose |  |  | 0.4 mL/body | | | | | 0.4 mL/body | | | | | 10^7^ cells/0.4 mL/body | | | | |
| Number of animals | |  | 6 | | | | | 6 | | | | | 6 | | | | |
| Color |  |  |  |  |  |  |  |  |  |  |  |  |  |  |  |  |  |
| Light yellow |  |  |  | 6 |  |  |  |  | 6 |  |  |  |  | 6 |  |  |  |
| pH |  |  |  |  |  |  |  |  |  |  |  |  |  |  |  |  |  |
| 7.0 |  |  |  | 0 |  |  |  |  | 1 |  |  |  |  | 0 |  |  |  |
| 8.0 |  |  |  | 0 |  |  |  |  | 1 |  |  |  |  | 0 |  |  |  |
| 8.5 |  |  |  | 4 |  |  |  |  | 3 |  |  |  |  | 3 |  |  |  |
| >9.0 |  |  |  | 2 |  |  |  |  | 1 |  |  |  |  | 3 |  |  |  |
| Protein |  |  |  |  |  |  |  |  |  |  |  |  |  |  |  |  |  |
| 30 mg/dL |  |  |  | 4 |  |  |  |  | 6 |  |  |  |  | 4 |  |  |  |
| 100 mg/dL |  |  |  | 2 |  |  |  |  | 0 |  |  |  |  | 2 |  |  |  |
| Glucose |  |  |  |  |  |  |  |  |  |  |  |  |  |  |  |  |  |
| Negative |  |  |  | 6 |  |  |  |  | 6 |  |  |  |  | 6 |  |  |  |
| Ketone body |  |  |  |  |  |  |  |  |  |  |  |  |  |  |  |  |  |
| Trace |  |  |  | 0 |  |  |  |  | 1 |  |  |  |  | 1 |  |  |  |
| Slight |  |  |  | 6 |  |  |  |  | 5 |  |  |  |  | 5 |  |  |  |
| Bilirubin |  |  |  |  |  |  |  |  |  |  |  |  |  |  |  |  |  |
| Negative |  |  |  | 4 |  |  |  |  | 3 |  |  |  |  | 3 |  |  |  |
| Slight |  |  |  | 2 |  |  |  |  | 3 |  |  |  |  | 3 |  |  |  |
| Occult blood |  |  |  |  |  |  |  |  |  |  |  |  |  |  |  |  |  |
| Negative |  |  |  | 6 |  |  |  |  | 6 |  |  |  |  | 6 |  |  |  |
| Urobilinogen |  |  |  |  |  |  |  |  |  |  |  |  |  |  |  |  |  |
| 0.1 E.U./dL |  |  |  | 1 |  |  |  |  | 4 |  |  |  |  | 2 |  |  |  |
| 1.0 E.U./dL |  |  |  | 5 |  |  |  |  | 2 |  |  |  |  | 4 |  |  |  |
|  |  |  |  |  |  |  |  |  |  |  |  |  |  |  |  |  |  |

Table S7. Urinalysis in female mice (27 or 28 days after administration)

|  |  |  |  |  |  |  |  |  |  |  |  |  |  |  |  |  |  |
| --- | --- | --- | --- | --- | --- | --- | --- | --- | --- | --- | --- | --- | --- | --- | --- | --- | --- |
| Group |  |  | Control (saline) | | | | | Control (culture media) | | | | | CellSaic | | | | |
| Dose |  |  | 0.4 mL/body | | | | | 0.4 mL/body | | | | | 10^7^ cells/0.4 mL/body | | | | |
| Number of animals | |  | 6 | | | | | 6 | | | | | 6 | | | | |
| Color |  |  |  |  |  |  |  |  |  |  |  |  |  |  |  |  |  |
| Light yellow |  |  |  | 6 |  |  |  |  | 6 |  |  |  |  | 6 |  |  |  |
| pH |  |  |  |  |  |  |  |  |  |  |  |  |  |  |  |  |  |
| 6.5 |  |  |  | 1 |  |  |  |  | 0 |  |  |  |  | 0 |  |  |  |
| 7.0 |  |  |  | 0 |  |  |  |  | 0 |  |  |  |  | 1 |  |  |  |
| 7.5 |  |  |  | 1 |  |  |  |  | 1 |  |  |  |  | 0 |  |  |  |
| 8.0 |  |  |  | 0 |  |  |  |  | 0 |  |  |  |  | 1 |  |  |  |
| 8.5 |  |  |  | 3 |  |  |  |  | 5 |  |  |  |  | 3 |  |  |  |
| >9.0 |  |  |  | 1 |  |  |  |  | 0 |  |  |  |  | 1 |  |  |  |
| Protein |  |  |  |  |  |  |  |  |  |  |  |  |  |  |  |  |  |
| Negative |  |  |  | 2 |  |  |  |  | 0 |  |  |  |  | 0 |  |  |  |
| Trace |  |  |  | 1 |  |  |  |  | 0 |  |  |  |  | 1 |  |  |  |
| 30 mg/dL |  |  |  | 3 |  |  |  |  | 5 |  |  |  |  | 4 |  |  |  |
| 100 mg/dL |  |  |  | 0 |  |  |  |  | 1 |  |  |  |  | 1 |  |  |  |
| Glucose |  |  |  |  |  |  |  |  |  |  |  |  |  |  |  |  |  |
| Negative |  |  |  | 6 |  |  |  |  | 6 |  |  |  |  | 6 |  |  |  |
| Ketone body |  |  |  |  |  |  |  |  |  |  |  |  |  |  |  |  |  |
| Negative |  |  |  | 1 |  |  |  |  | 0 |  |  |  |  | 0 |  |  |  |
| Trace |  |  |  | 2 |  |  |  |  | 1 |  |  |  |  | 3 |  |  |  |
| Slight |  |  |  | 3 |  |  |  |  | 5 |  |  |  |  | 3 |  |  |  |
| Bilirubin |  |  |  |  |  |  |  |  |  |  |  |  |  |  |  |  |  |
| Negative |  |  |  | 3 |  |  |  |  | 0 |  |  |  |  | 2 |  |  |  |
| Slight |  |  |  | 3 |  |  |  |  | 5 |  |  |  |  | 4 |  |  |  |
| Moderate |  |  |  | 0 |  |  |  |  | 1 |  |  |  |  | 0 |  |  |  |
| Occult blood |  |  |  |  |  |  |  |  |  |  |  |  |  |  |  |  |  |
| Negative |  |  |  | 6 |  |  |  |  | 6 |  |  |  |  | 6 |  |  |  |
| Urobilinogen |  |  |  |  |  |  |  |  |  |  |  |  |  |  |  |  |  |
| 0.1 E.U./dL |  |  |  | 4 |  |  |  |  | 2 |  |  |  |  | 1 |  |  |  |
| 1.0 E.U./dL |  |  |  | 2 |  |  |  |  | 4 |  |  |  |  | 5 |  |  |  |
|  |  |  |  |  |  |  |  |  |  |  |  |  |  |  |  |  |  |

Table S8. Urinalysis in male mice (55 or 56 days after administration)

|  |  |  |  |  |  |  |  |  |  |  |  |  |  |  |  |  |  |
| --- | --- | --- | --- | --- | --- | --- | --- | --- | --- | --- | --- | --- | --- | --- | --- | --- | --- |
| Group |  |  | Control (saline) | | | | | Control (culture media) | | | | | CellSaic | | | | |
| Dose |  |  | 0.4 mL/body | | | | | 0.4 mL/body | | | | | 10^7^ cells/0.4 mL/body | | | | |
| Number of animals | |  | 6 | | | | | 6 | | | | | 6 | | | | |
| Color |  |  |  |  |  |  |  |  |  |  |  |  |  |  |  |  |  |
| Light yellow |  |  |  | 6 |  |  |  |  | 6 |  |  |  |  | 6 |  |  |  |
| pH |  |  |  |  |  |  |  |  |  |  |  |  |  |  |  |  |  |
| 8.0 |  |  |  | 1 |  |  |  |  | 0 |  |  |  |  | 0 |  |  |  |
| 8.5 |  |  |  | 5 |  |  |  |  | 6 |  |  |  |  | 4 |  |  |  |
| >9.0 |  |  |  | 0 |  |  |  |  | 0 |  |  |  |  | 2 |  |  |  |
| Protein |  |  |  |  |  |  |  |  |  |  |  |  |  |  |  |  |  |
| Trace |  |  |  | 0 |  |  |  |  | 1 |  |  |  |  | 2 |  |  |  |
| 30 mg/dL |  |  |  | 5 |  |  |  |  | 4 |  |  |  |  | 3 |  |  |  |
| 100 mg/dL |  |  |  | 1 |  |  |  |  | 1 |  |  |  |  | 1 |  |  |  |
| Glucose |  |  |  |  |  |  |  |  |  |  |  |  |  |  |  |  |  |
| Negative |  |  |  | 6 |  |  |  |  | 6 |  |  |  |  | 6 |  |  |  |
| Ketone body |  |  |  |  |  |  |  |  |  |  |  |  |  |  |  |  |  |
| Trace |  |  |  | 0 |  |  |  |  | 1 |  |  |  |  | 2 |  |  |  |
| Slight |  |  |  | 6 |  |  |  |  | 5 |  |  |  |  | 4 |  |  |  |
| Bilirubin |  |  |  |  |  |  |  |  |  |  |  |  |  |  |  |  |  |
| Negative |  |  |  | 4 |  |  |  |  | 1 |  |  |  |  | 3 |  |  |  |
| Slight |  |  |  | 2 |  |  |  |  | 5 |  |  |  |  | 3 |  |  |  |
| Occult blood |  |  |  |  |  |  |  |  |  |  |  |  |  |  |  |  |  |
| Negative |  |  |  | 6 |  |  |  |  | 6 |  |  |  |  | 6 |  |  |  |
| Urobilinogen |  |  |  |  |  |  |  |  |  |  |  |  |  |  |  |  |  |
| 0.1 E.U./dL |  |  |  | 4 |  |  |  |  | 2 |  |  |  |  | 5 |  |  |  |
| 1.0 E.U./dL |  |  |  | 2 |  |  |  |  | 4 |  |  |  |  | 1 |  |  |  |
|  |  |  |  |  |  |  |  |  |  |  |  |  |  |  |  |  |  |

Table S9. Urinalysis in female mice (55 or 56 days after administration)

|  |  |  |  |  |  |  |  |  |  |  |  |  |  |  |  |  |  |
| --- | --- | --- | --- | --- | --- | --- | --- | --- | --- | --- | --- | --- | --- | --- | --- | --- | --- |
| Group |  |  | Control (saline) | | | | | Control (culture media) | | | | | CellSaic | | | | |
| Dose |  |  | 0.4 mL/body | | | | | 0.4 mL/body | | | | | 10^7^ cells/0.4 mL/body | | | | |
| Number of animals | |  | 6 | | | | | 6 | | | | | 6 | | | | |
| Color |  |  |  |  |  |  |  |  |  |  |  |  |  |  |  |  |  |
| Light yellow |  |  |  | 6 |  |  |  |  | 6 |  |  |  |  | 6 |  |  |  |
| pH |  |  |  |  |  |  |  |  |  |  |  |  |  |  |  |  |  |
| 7.5 |  |  |  | 0 |  |  |  |  | 1 |  |  |  |  | 0 |  |  |  |
| 8.5 |  |  |  | 4 |  |  |  |  | 4 |  |  |  |  | 4 |  |  |  |
| >9.0 |  |  |  | 2 |  |  |  |  | 1 |  |  |  |  | 2 |  |  |  |
| Protein |  |  |  |  |  |  |  |  |  |  |  |  |  |  |  |  |  |
| Negative |  |  |  | 0 |  |  |  |  | 1 |  |  |  |  | 2 |  |  |  |
| Trace |  |  |  | 0 |  |  |  |  | 3 |  |  |  |  | 1 |  |  |  |
| 30 mg/dL |  |  |  | 4 |  |  |  |  | 1 |  |  |  |  | 1 |  |  |  |
| 100 mg/dL |  |  |  | 2 |  |  |  |  | 1 |  |  |  |  | 2 |  |  |  |
| Glucose |  |  |  |  |  |  |  |  |  |  |  |  |  |  |  |  |  |
| Negative |  |  |  | 6 |  |  |  |  | 6 |  |  |  |  | 6 |  |  |  |
| Ketone body |  |  |  |  |  |  |  |  |  |  |  |  |  |  |  |  |  |
| Negative |  |  |  | 0 |  |  |  |  | 1 |  |  |  |  | 0 |  |  |  |
| Trace |  |  |  | 1 |  |  |  |  | 4 |  |  |  |  | 4 |  |  |  |
| Slight |  |  |  | 5 |  |  |  |  | 1 |  |  |  |  | 2 |  |  |  |
| Bilirubin |  |  |  |  |  |  |  |  |  |  |  |  |  |  |  |  |  |
| Negative |  |  |  | 0 |  |  |  |  | 3 |  |  |  |  | 3 |  |  |  |
| Slight |  |  |  | 4 |  |  |  |  | 3 |  |  |  |  | 3 |  |  |  |
| Moderate |  |  |  | 2 |  |  |  |  | 0 |  |  |  |  | 0 |  |  |  |
| Occult blood |  |  |  |  |  |  |  |  |  |  |  |  |  |  |  |  |  |
| Negative |  |  |  | 6 |  |  |  |  | 6 |  |  |  |  | 6 |  |  |  |
| Urobilinogen |  |  |  |  |  |  |  |  |  |  |  |  |  |  |  |  |  |
| 0.1 E.U./dL |  |  |  | 0 |  |  |  |  | 5 |  |  |  |  | 5 |  |  |  |
| 1.0 E.U./dL |  |  |  | 6 |  |  |  |  | 1 |  |  |  |  | 1 |  |  |  |
|  |  |  |  |  |  |  |  |  |  |  |  |  |  |  |  |  |  |

Table S10. Hematological findings in male mice (28 days after administration)

|  |  |  |  |  |  |  |  |  |  |  |  |  |  |  |  |  |
| --- | --- | --- | --- | --- | --- | --- | --- | --- | --- | --- | --- | --- | --- | --- | --- | --- |
| Group |  | Control (saline) |  |  |  |  | Control (culture media) |  |  |  |  | CellSaic |  |  |  |  |
| Dose |  | 0.4 mL/body |  |  |  |  | 0.4 mL/body |  |  |  |  | 10^7^ cells/0.4 mL/body |  |  |  |  |
| Number of animals |  | 6 | | | | | 6 | | | | | 6 | | | | |
| RBC | (10^4^/μL) | 846 | ± | 28 |  |  | 843 | ± | 7 |  |  | 857 | ± | 41 |  |  |
| HGB | (g/dL) | 13.0 | ± | 0.3 |  |  | 13.0 | ± | 0.2 |  |  | 13.1 | ± | 0.5 |  |  |
| HCT | (%) | 41.1 | ± | 1.0 |  |  | 41.1 | ± | 0.5 |  |  | 42.0 | ± | 1.5 |  |  |
| MCV | (fL) | 48.7 | ± | 0.7 |  |  | 48.8 | ± | 0.3 |  |  | 49.0 | ± | 1.1 |  |  |
| MCH | (pg) | 15.4 | ± | 0.2 |  |  | 15.4 | ± | 0.1 |  |  | 15.3 | ± | 0.3 |  |  |
| MCHC | (g/dL) | 31.7 | ± | 0.3 |  |  | 31.6 | ± | 0.1 |  |  | 31.3 | ± | 0.5 |  |  |
| PLT | (10^4^/μL) | 185.4 | ± | 32.4 |  |  | 195.2 | ± | 11.6 |  |  | 210.6 | ± | 17.9 |  |  |
| RET | (10^4^/μL) | 37.21 | ± | 3.33 |  |  | 33.92 | ± | 1.58 |  |  | 42.91 | ± | 17.87 |  |  |
| RET | (%) | 4.40 | ± | 0.28 |  |  | 4.03 | ± | 0.19 | # |  | 5.07 | ± | 2.36 |  |  |
| WBC | (10^2^/μL) | 5.0 | ± | 2.0 |  |  | 5.0 | ± | 1.5 |  |  | 4.0 | ± | 1.4 |  |  |
| Differential leukocyte (10^2^/μL) | |  |  |  |  |  |  |  |  |  |  |  |  |  |  |  |
| Lymphocyte |  | 1.5 | ± | 1.6 |  |  | 1.2 | ± | 0.6 |  |  | 1.0 | ± | 0.7 |  |  |
| Neutrophil |  | 2.7 | ± | 1.1 |  |  | 3.4 | ± | 1.2 |  |  | 2.8 | ± | 0.8 |  |  |
| Eosinophil |  | 0.7 | ± | 1.3 |  |  | 0.2 | ± | 0.1 |  |  | 0.1 | ± | 0.1 |  |  |
| Basophil |  | 0.0 | ± | 0.0 |  |  | 0.0 | ± | 0.0 |  |  | 0.0 | ± | 0.0 |  |  |
| Monocyte |  | 0.1 | ± | 0.1 |  |  | 0.2 | ± | 0.2 |  |  | 0.1 | ± | 0.2 |  |  |
| Differential leukocyte (%) | |  |  |  |  |  |  |  |  |  |  |  |  |  |  |  |
| Lymphocyte |  | 25.7 | ± | 14.7 |  |  | 23.9 | ± | 9.0 |  |  | 22.2 | ± | 8.4 |  |  |
| Neutrophil |  | 62.7 | ± | 28.0 |  |  | 67.8 | ± | 8.6 |  |  | 71.8 | ± | 8.6 |  |  |
| Eosinophil |  | 9.8 | ± | 14.1 |  |  | 3.5 | ± | 1.3 |  |  | 2.8 | ± | 1.9 |  |  |
| Basophil |  | 0.0 | ± | 0.0 |  |  | 0.4 | ± | 1.1 |  |  | 0.0 | ± | 0.0 |  |  |
| Monocyte |  | 1.9 | ± | 2.2 |  |  | 4.4 | ± | 3.8 |  |  | 3.3 | ± | 3.4 |  |  |
| Each value shows mean ± S.D. | |  |  |  |  |  |  |  |  |  |  |  |  |  |  |  |
| Significantly different from the control (saline) group at 0.4 mL/body (#: p<0.05 by Steel-Dwass test). | | | | | | | | | |  |  |  |  |  |  |  |
|  |  |  |  |  |  |  |  |  |  |  |  |  |  |  |  |  |

Table S11. Hematological findings in female mice (28 days after administration)

|  |  |  |  |  |  |  |  |  |  |  |  |  |  |  |  |  |
| --- | --- | --- | --- | --- | --- | --- | --- | --- | --- | --- | --- | --- | --- | --- | --- | --- |
| Group |  | Control (saline) |  |  |  |  | Control (culture media) |  |  |  |  | CellSaic |  |  |  |  |
| Dose |  | 0.4 mL/body |  |  |  |  | 0.4 mL/body |  |  |  |  | 10^7^ cells/0.4 mL/body |  |  |  |  |
| Number of animals |  | 6 | | | | | 6 | | | | | 6 | | | | |
| RBC | (10^4^/μL) | 841 | ± | 38 |  |  | 843 | ± | 23 |  |  | 843 | ± | 20 |  |  |
| HGB | (g/dL) | 13.2 | ± | 0.5 |  |  | 13.2 | ± | 0.5 |  |  | 13.2 | ± | 0.3 |  |  |
| HCT | (%) | 41.2 | ± | 1.2 |  |  | 41.0 | ± | 1.3 |  |  | 41.2 | ± | 0.6 |  |  |
| MCV | (fL) | 49.1 | ± | 1.0 |  |  | 48.5 | ± | 0.7 |  |  | 48.9 | ± | 0.8 |  |  |
| MCH | (pg) | 15.7 | ± | 0.3 |  |  | 15.6 | ± | 0.3 |  |  | 15.7 | ± | 0.1 |  |  |
| MCHC | (g/dL) | 32.1 | ± | 0.4 |  |  | 32.1 | ± | 0.4 |  |  | 32.1 | ± | 0.4 |  |  |
| PLT | (10^4^/μL) | 152.8 | ± | 27.6 |  |  | 156.6 | ± | 9.2 |  |  | 150.9 | ± | 14.1 |  |  |
| RET | (10^4^/μL) | 43.34 | ± | 6.13 |  |  | 39.38 | ± | 3.26 |  |  | 41.63 | ± | 3.03 |  |  |
| RET | (%) | 5.16 | ± | 0.73 |  |  | 4.67 | ± | 0.34 |  |  | 4.95 | ± | 0.40 |  |  |
| WBC | (10^2^/μL) | 6.4 | ± | 2.7 |  |  | 6.6 | ± | 2.5 |  |  | 6.4 | ± | 2.9 |  |  |
| Differential leukocyte (10^2^/μL) | |  |  |  |  |  |  |  |  |  |  |  |  |  |  |  |
| Lymphocyte |  | 0.8 | ± | 0.4 |  |  | 1.1 | ± | 0.3 |  |  | 1.4 | ± | 1.1 |  |  |
| Neutrophil |  | 5.4 | ± | 2.5 |  |  | 5.1 | ± | 2.2 |  |  | 4.7 | ± | 2.8 |  |  |
| Eosinophil |  | 0.1 | ± | 0.1 |  |  | 0.1 | ± | 0.0 |  |  | 0.1 | ± | 0.1 |  |  |
| Basophil |  | 0.0 | ± | 0.0 |  |  | 0.0 | ± | 0.0 |  |  | 0.0 | ± | 0.0 |  |  |
| Monocyte |  | 0.2 | ± | 0.1 |  |  | 0.2 | ± | 0.1 |  |  | 0.2 | ± | 0.2 |  |  |
| Differential leukocyte (%) | |  |  |  |  |  |  |  |  |  |  |  |  |  |  |  |
| Lymphocyte |  | 13.4 | ± | 6.2 |  |  | 19.0 | ± | 7.4 |  |  | 22.2 | ± | 20.1 |  |  |
| Neutrophil |  | 82.8 | ± | 6.6 |  |  | 75.5 | ± | 9.5 |  |  | 72.0 | ± | 23.6 |  |  |
| Eosinophil |  | 1.1 | ± | 1.0 |  |  | 1.2 | ± | 0.6 |  |  | 2.2 | ± | 0.4 | * |  |
| Basophil |  | 0.0 | ± | 0.0 |  |  | 0.0 | ± | 0.0 |  |  | 0.0 | ± | 0.0 |  |  |
| Monocyte |  | 2.7 | ± | 2.4 |  |  | 4.4 | ± | 3.0 |  |  | 3.7 | ± | 4.0 |  |  |
| Each value shows mean ± S.D. | |  |  |  |  |  |  |  |  |  |  |  |  |  |  |  |
| Significantly different from the control (saline) group at 0.4 mL/body (*: p<0.05 by Tukey-Kramer test). | | | | | | | | | | |  |  |  |  |  |  |
|  |  |  |  |  |  |  |  |  |  |  |  |  |  |  |  |  |

Table S12. Hematological findings in male mice (56 days after administration)

|  |  |  |  |  |  |  |  |  |  |  |  |  |  |  |  |  |
| --- | --- | --- | --- | --- | --- | --- | --- | --- | --- | --- | --- | --- | --- | --- | --- | --- |
| Group |  | Control (saline) |  |  |  |  | Control (culture media) |  |  |  |  | CellSaic |  |  |  |  |
| Dose |  | 0.4 mL/body |  |  |  |  | 0.4 mL/body |  |  |  |  | 10^7^ cells/0.4 mL/body |  |  |  |  |
| Number of animals |  | 6 | | | | | 6 | | | | | 6 | | | | |
| RBC | (10^4^/μL) | 832 | ± | 20 |  |  | 837 | ± | 19 |  |  | 837 | ± | 20 |  |  |
| HGB | (g/dL) | 12.7 | ± | 0.3 |  |  | 12.8 | ± | 0.3 |  |  | 12.7 | ± | 0.3 |  |  |
| HCT | (%) | 40.6 | ± | 1.0 |  |  | 40.9 | ± | 1.0 |  |  | 40.5 | ± | 0.8 |  |  |
| MCV | (fL) | 48.8 | ± | 0.3 |  |  | 48.8 | ± | 0.5 |  |  | 48.4 | ± | 0.5 |  |  |
| MCH | (pg) | 15.3 | ± | 0.1 |  |  | 15.3 | ± | 0.1 |  |  | 15.2 | ± | 0.1 |  |  |
| MCHC | (g/dL) | 31.3 | ± | 0.3 |  |  | 31.4 | ± | 0.4 |  |  | 31.4 | ± | 0.3 |  |  |
| PLT | (10^4^/μL) | 188.2 | ± | 45.7 |  |  | 202.2 | ± | 10.4 |  |  | 207.5 | ± | 10.3 |  |  |
| RET | (10^4^/μL) | 33.94 | ± | 2.32 |  |  | 34.66 | ± | 1.73 |  |  | 31.81 | ± | 1.23 | * |  |
| RET | (%) | 4.08 | ± | 0.25 |  |  | 4.14 | ± | 0.17 |  |  | 3.80 | ± | 0.13 | * |  |
| WBC | (10^2^/μL) | 4.7 | ± | 0.6 |  |  | 5.5 | ± | 1.1 |  |  | 6.1 | ± | 1.6 |  |  |
| Differential leukocyte (10^2^/μL) | |  |  |  |  |  |  |  |  |  |  |  |  |  |  |  |
| Lymphocyte |  | 1.2 | ± | 0.4 |  |  | 1.3 | ± | 0.3 |  |  | 1.5 | ± | 0.5 |  |  |
| Neutrophil |  | 3.0 | ± | 1.1 |  |  | 3.9 | ± | 0.9 |  |  | 4.4 | ± | 1.1 |  |  |
| Eosinophil |  | 0.3 | ± | 0.5 |  |  | 0.2 | ± | 0.1 |  |  | 0.1 | ± | 0.0 |  |  |
| Basophil |  | 0.0 | ± | 0.0 |  |  | 0.0 | ± | 0.0 |  |  | 0.1 | ± | 0.2 |  |  |
| Monocyte |  | 0.2 | ± | 0.1 |  |  | 0.1 | ± | 0.1 |  |  | 0.1 | ± | 0.1 |  |  |
| Differential leukocyte (%) | |  |  |  |  |  |  |  |  |  |  |  |  |  |  |  |
| Lymphocyte |  | 25.0 | ± | 9.3 |  |  | 23.9 | ± | 5.0 |  |  | 23.7 | ± | 3.1 |  |  |
| Neutrophil |  | 62.9 | ± | 19.4 |  |  | 71.5 | ± | 6.1 |  |  | 72.1 | ± | 3.7 |  |  |
| Eosinophil |  | 7.2 | ± | 11.4 |  |  | 2.7 | ± | 1.4 |  |  | 2.2 | ± | 1.4 |  |  |
| Basophil |  | 0.0 | ± | 0.0 |  |  | 0.0 | ± | 0.0 |  |  | 0.9 | ± | 2.2 |  |  |
| Monocyte |  | 4.8 | ± | 3.4 |  |  | 1.8 | ± | 1.6 |  |  | 1.2 | ± | 1.9 |  |  |
| Each value shows mean ± S.D. | |  |  |  |  |  |  |  |  |  |  |  |  |  |  |  |
| Significantly different from the control (culture media) group at 0.4 mL/body (*: p<0.05 by Tukey-Kramer test). | | | | | | | | | | | |  |  |  |  |  |
|  |  |  |  |  |  |  |  |  |  |  |  |  |  |  |  |  |

Table S13. Hematological findings in female mice (56 days after administration)

|  |  |  |  |  |  |  |  |  |  |  |  |  |  |  |  |  |
| --- | --- | --- | --- | --- | --- | --- | --- | --- | --- | --- | --- | --- | --- | --- | --- | --- |
| Group |  | Control (saline) |  |  |  |  | Control (culture media) |  |  |  |  | CellSaic |  |  |  |  |
| Dose |  | 0.4 mL/body |  |  |  |  | 0.4 mL/body |  |  |  |  | 10^7^ cells/0.4 mL/body |  |  |  |  |
| Number of animals |  | 6 | | | | | 6 | | | | | 6 | | | | |
| RBC | (10^4^/μL) | 832 | ± | 20 |  |  | 837 | ± | 19 |  |  | 837 | ± | 20 |  |  |
| HGB | (g/dL) | 12.7 | ± | 0.3 |  |  | 12.8 | ± | 0.3 |  |  | 12.7 | ± | 0.3 |  |  |
| HCT | (%) | 40.6 | ± | 1.0 |  |  | 40.9 | ± | 1.0 |  |  | 40.5 | ± | 0.8 |  |  |
| MCV | (fL) | 48.8 | ± | 0.3 |  |  | 48.8 | ± | 0.5 |  |  | 48.4 | ± | 0.5 |  |  |
| MCH | (pg) | 15.3 | ± | 0.1 |  |  | 15.3 | ± | 0.1 |  |  | 15.2 | ± | 0.1 |  |  |
| MCHC | (g/dL) | 31.3 | ± | 0.3 |  |  | 31.4 | ± | 0.4 |  |  | 31.4 | ± | 0.3 |  |  |
| PLT | (10^4^/μL) | 188.2 | ± | 45.7 |  |  | 202.2 | ± | 10.4 |  |  | 207.5 | ± | 10.3 |  |  |
| RET | (10^4^/μL) | 33.94 | ± | 2.32 |  |  | 34.66 | ± | 1.73 |  |  | 31.81 | ± | 1.23 | * |  |
| RET | (%) | 4.08 | ± | 0.25 |  |  | 4.14 | ± | 0.17 |  |  | 3.80 | ± | 0.13 | * |  |
| WBC | (10^2^/μL) | 4.7 | ± | 0.6 |  |  | 5.5 | ± | 1.1 |  |  | 6.1 | ± | 1.6 |  |  |
| Differential leukocyte (10^2^/μL) | |  |  |  |  |  |  |  |  |  |  |  |  |  |  |  |
| Lymphocyte |  | 1.2 | ± | 0.4 |  |  | 1.3 | ± | 0.3 |  |  | 1.5 | ± | 0.5 |  |  |
| Neutrophil |  | 3.0 | ± | 1.1 |  |  | 3.9 | ± | 0.9 |  |  | 4.4 | ± | 1.1 |  |  |
| Eosinophil |  | 0.3 | ± | 0.5 |  |  | 0.2 | ± | 0.1 |  |  | 0.1 | ± | 0.0 |  |  |
| Basophil |  | 0.0 | ± | 0.0 |  |  | 0.0 | ± | 0.0 |  |  | 0.1 | ± | 0.2 |  |  |
| Monocyte |  | 0.2 | ± | 0.1 |  |  | 0.1 | ± | 0.1 |  |  | 0.1 | ± | 0.1 |  |  |
| Differential leukocyte (%) | |  |  |  |  |  |  |  |  |  |  |  |  |  |  |  |
| Lymphocyte |  | 25.0 | ± | 9.3 |  |  | 23.9 | ± | 5.0 |  |  | 23.7 | ± | 3.1 |  |  |
| Neutrophil |  | 62.9 | ± | 19.4 |  |  | 71.5 | ± | 6.1 |  |  | 72.1 | ± | 3.7 |  |  |
| Eosinophil |  | 7.2 | ± | 11.4 |  |  | 2.7 | ± | 1.4 |  |  | 2.2 | ± | 1.4 |  |  |
| Basophil |  | 0.0 | ± | 0.0 |  |  | 0.0 | ± | 0.0 |  |  | 0.9 | ± | 2.2 |  |  |
| Monocyte |  | 4.8 | ± | 3.4 |  |  | 1.8 | ± | 1.6 |  |  | 1.2 | ± | 1.9 |  |  |
| Each value shows mean ± S.D. | |  |  |  |  |  |  |  |  |  |  |  |  |  |  |  |
| Significantly different from the control (culture media) group at 0.4 mL/body (*: p<0.05 by Tukey-Kramer test). | | | | | | | | | | | |  |  |  |  |  |
|  |  |  |  |  |  |  |  |  |  |  |  |  |  |  |  |  |

Table S14. Blood chemistry in male mice (28 days after administration)

|  |  |  |  |  |  |  |  |  |  |  |  |  |  |  |  |  |
| --- | --- | --- | --- | --- | --- | --- | --- | --- | --- | --- | --- | --- | --- | --- | --- | --- |
| Group |  | Control (saline) |  |  |  |  | Control (culture media) |  |  |  |  | CellSaic |  |  |  |  |
| Dose |  | 0.4 mL/body |  |  |  |  | 0.4 mL/body |  |  |  |  | 10^7^ cells/0.4 mL/body |  |  |  |  |
| Number of animals | | 6 | | | | | 6 | | | | | 6 | | | | |
| AST | (IU/L) | 39.5 | ± | 4.7 |  |  | 41.0 | ± | 3.2 |  |  | 40.7 | ± | 6.2 |  |  |
| ALT | (IU/L) | 15.8 | ± | 1.0 |  |  | 16.4 | ± | 1.6 |  |  | 16.1 | ± | 2.5 |  |  |
| ALP | (IU/L) | 212.3 | ± | 13.8 |  |  | 219.8 | ± | 9.8 |  |  | 217.1 | ± | 13.3 |  |  |
| TP | (g/dL) | 4.79 | ± | 0.15 |  |  | 4.76 | ± | 0.13 |  |  | 4.83 | ± | 0.21 |  |  |
| Alb | (g/dL) | 2.09 | ± | 0.07 |  |  | 2.07 | ± | 0.06 |  |  | 2.10 | ± | 0.09 |  |  |
| A/G |  | 0.78 | ± | 0.02 |  |  | 0.77 | ± | 0.01 |  |  | 0.77 | ± | 0.01 |  |  |
| T-Bil | (mg/dL) | 0.21 | ± | 0.02 |  |  | 0.20 | ± | 0.02 |  |  | 0.21 | ± | 0.03 |  |  |
| UN | (mg/dL) | 24.2 | ± | 2.9 |  |  | 23.7 | ± | 3.9 |  |  | 24.1 | ± | 1.2 |  |  |
| CRE | (mg/dL) | 0.13 | ± | 0.04 |  |  | 0.13 | ± | 0.04 |  |  | 0.10 | ± | 0.01 |  |  |
| Glu | (mg/dL) | 184.8 | ± | 17.4 |  |  | 184.3 | ± | 13.0 |  |  | 197.9 | ± | 15.5 |  |  |
| T-Cho | (mg/dL) | 106.0 | ± | 8.1 |  |  | 106.8 | ± | 5.9 |  |  | 106.8 | ± | 8.9 |  |  |
| TG | (mg/dL) | 39.0 | ± | 18.2 |  |  | 34.7 | ± | 23.3 |  |  | 40.7 | ± | 27.9 |  |  |
| Na | (mEq/L) | 150.6 | ± | 1.3 |  |  | 151.0 | ± | 0.7 |  |  | 151.4 | ± | 1.0 |  |  |
| K | (mEq/L) | 4.98 | ± | 0.17 |  |  | 4.91 | ± | 0.23 |  |  | 4.99 | ± | 0.32 |  |  |
| Cl | (mEq/L) | 115.9 | ± | 1.2 |  |  | 116.0 | ± | 1.3 |  |  | 116.8 | ± | 2.8 |  |  |
| Ca | (mg/dL) | 9.2 | ± | 0.2 |  |  | 9.1 | ± | 0.2 |  |  | 9.2 | ± | 0.3 |  |  |
| IP | (mg/dL) | 5.9 | ± | 0.3 |  |  | 5.7 | ± | 0.6 |  |  | 6.0 | ± | 1.2 |  |  |
| Each value shows mean ± S.D. | | |  |  |  |  |  |  |  |  |  |  |  |  |  |  |
|  |  |  |  |  |  |  |  |  |  |  |  |  |  |  |  |  |

Table S15. Blood chemistry in female mice (28 days after administration)

|  |  |  |  |  |  |  |  |  |  |  |  |  |  |  |  |  |
| --- | --- | --- | --- | --- | --- | --- | --- | --- | --- | --- | --- | --- | --- | --- | --- | --- |
| Group |  | Control (saline) |  |  |  |  | Control (culture media) |  |  |  |  | CellSaic |  |  |  |  |
| Dose |  | 0.4 mL/body |  |  |  |  | 0.4 mL/body |  |  |  |  | 10^7^ cells/0.4 mL/body |  |  |  |  |
| Number of animals | | 6 | | | | | 6 | | | | | 6 | | | | |
| AST | (IU/L) | 46.3 | ± | 5.2 |  |  | 51.8 | ± | 7.8 |  |  | 50.8 | ± | 11.5 |  |  |
| ALT | (IU/L) | 15.3 | ± | 1.3 |  |  | 18.1 | ± | 2.1 |  |  | 16.7 | ± | 2.5 |  |  |
| ALP | (IU/L) | 353.8 | ± | 40.7 |  |  | 312.9 | ± | 28.1 |  |  | 340.4 | ± | 45.7 |  |  |
| TP | (g/dL) | 4.67 | ± | 0.16 |  |  | 4.59 | ± | 0.07 |  |  | 4.65 | ± | 0.13 |  |  |
| Alb | (g/dL) | 2.23 | ± | 0.05 |  |  | 2.17 | ± | 0.05 |  |  | 2.23 | ± | 0.06 |  |  |
| A/G |  | 0.91 | ± | 0.03 |  |  | 0.90 | ± | 0.03 |  |  | 0.92 | ± | 0.02 |  |  |
| T-Bil | (mg/dL) | 0.15 | ± | 0.02 |  |  | 0.16 | ± | 0.01 |  |  | 0.17 | ± | 0.03 |  |  |
| UN | (mg/dL) | 24.8 | ± | 3.1 |  |  | 23.8 | ± | 2.8 |  |  | 25.0 | ± | 3.3 |  |  |
| CRE | (mg/dL) | 0.14 | ± | 0.03 |  |  | 0.15 | ± | 0.02 |  |  | 0.12 | ± | 0.01 |  |  |
| Glu | (mg/dL) | 172.9 | ± | 37.8 |  |  | 181.4 | ± | 27.6 |  |  | 173.4 | ± | 32.9 |  |  |
| T-Cho | (mg/dL) | 85.0 | ± | 12.1 |  |  | 82.2 | ± | 5.0 |  |  | 83.2 | ± | 5.3 |  |  |
| TG | (mg/dL) | 69.4 | ± | 31.1 |  |  | 43.8 | ± | 38.9 |  |  | 37.3 | ± | 14.6 |  |  |
| Na | (mEq/L) | 147.4 | ± | 2.1 |  |  | 146.2 | ± | 1.0 |  |  | 147.5 | ± | 0.9 |  |  |
| K | (mEq/L) | 4.44 | ± | 0.24 |  |  | 4.58 | ± | 0.12 |  |  | 4.50 | ± | 0.30 |  |  |
| Cl | (mEq/L) | 116.4 | ± | 1.3 |  |  | 116.4 | ± | 2.1 |  |  | 116.6 | ± | 1.6 |  |  |
| Ca | (mg/dL) | 9.1 | ± | 0.2 |  |  | 9.0 | ± | 0.2 |  |  | 9.0 | ± | 0.1 |  |  |
| IP | (mg/dL) | 5.7 | ± | 0.8 |  |  | 5.3 | ± | 0.7 |  |  | 5.3 | ± | 0.8 |  |  |
| Each value shows mean ± S.D. | | |  |  |  |  |  |  |  |  |  |  |  |  |  |  |
|  |  |  |  |  |  |  |  |  |  |  |  |  |  |  |  |  |

Table S16. Blood chemistry in male mice (56 days after administration)

|  |  |  |  |  |  |  |  |  |  |  |  |  |  |  |  |  |
| --- | --- | --- | --- | --- | --- | --- | --- | --- | --- | --- | --- | --- | --- | --- | --- | --- |
| Group |  | Control (saline) |  |  |  |  | Control (culture media) |  |  |  |  | CellSaic |  |  |  |  |
| Dose |  | 0.4 mL/body |  |  |  |  | 0.4 mL/body |  |  |  |  | 10^7^ cells/0.4 mL/body |  |  |  |  |
| Number of animals | | 6 | | | | | 6 | | | | | 6 | | | | |
| AST | (IU/L) | 40.4 | ± | 3.9 |  |  | 39.2 | ± | 2.6 |  |  | 40.6 | ± | 5.0 |  |  |
| ALT | (IU/L) | 18.4 | ± | 1.9 |  |  | 17.0 | ± | 2.3 |  |  | 17.8 | ± | 1.5 |  |  |
| ALP | (IU/L) | 169.0 | ± | 22.0 |  |  | 180.8 | ± | 21.4 |  |  | 182.2 | ± | 31.3 |  |  |
| TP | (g/dL) | 4.51 | ± | 0.23 |  |  | 4.52 | ± | 0.07 |  |  | 4.46 | ± | 0.09 |  |  |
| Alb | (g/dL) | 1.98 | ± | 0.09 |  |  | 1.97 | ± | 0.05 |  |  | 1.96 | ± | 0.04 |  |  |
| A/G |  | 0.78 | ± | 0.01 |  |  | 0.77 | ± | 0.01 |  |  | 0.79 | ± | 0.01 |  |  |
| T-Bil | (mg/dL) | 0.17 | ± | 0.02 |  |  | 0.19 | ± | 0.03 |  |  | 0.18 | ± | 0.02 |  |  |
| UN | (mg/dL) | 23.4 | ± | 1.3 |  |  | 21.7 | ± | 0.9 |  |  | 21.9 | ± | 2.7 |  |  |
| CRE | (mg/dL) | 0.12 | ± | 0.01 |  |  | 0.12 | ± | 0.02 |  |  | 0.11 | ± | 0.01 |  |  |
| Glu | (mg/dL) | 180.6 | ± | 19.1 |  |  | 196.2 | ± | 22.4 |  |  | 190.6 | ± | 22.3 |  |  |
| T-Cho | (mg/dL) | 95.0 | ± | 4.0 |  |  | 90.8 | ± | 9.6 |  |  | 89.3 | ± | 6.1 |  |  |
| TG | (mg/dL) | 51.6 | ± | 33.9 |  |  | 39.7 | ± | 16.4 |  |  | 32.1 | ± | 11.6 |  |  |
| Na | (mEq/L) | 152.1 | ± | 1.3 |  |  | 151.5 | ± | 1.4 |  |  | 152.2 | ± | 2.1 |  |  |
| K | (mEq/L) | 5.23 | ± | 0.39 |  |  | 4.96 | ± | 0.32 |  |  | 5.31 | ± | 0.35 |  |  |
| Cl | (mEq/L) | 117.0 | ± | 2.0 |  |  | 116.3 | ± | 0.8 |  |  | 117.5 | ± | 2.9 |  |  |
| Ca | (mg/dL) | 9.2 | ± | 0.2 |  |  | 8.9 | ± | 0.1 |  |  | 9.0 | ± | 0.2 |  |  |
| IP | (mg/dL) | 6.2 | ± | 0.8 |  |  | 6.2 | ± | 0.8 |  |  | 6.0 | ± | 0.4 |  |  |
| Each value shows mean ± S.D. | | |  |  |  |  |  |  |  |  |  |  |  |  |  |  |
|  |  |  |  |  |  |  |  |  |  |  |  |  |  |  |  |  |

Table S17. Blood chemistry in female mice (56 days after administration)

|  |  |  |  |  |  |  |  |  |  |  |  |  |  |  |  |  |
| --- | --- | --- | --- | --- | --- | --- | --- | --- | --- | --- | --- | --- | --- | --- | --- | --- |
| Group |  | Control (saline) |  |  |  |  | Control (culture media) |  |  |  |  | CellSaic |  |  |  |  |
| Dose |  | 0.4 mL/body |  |  |  |  | 0.4 mL/body |  |  |  |  | 10^7^ cells/0.4 mL/body |  |  |  |  |
| Number of animals | | 6 | | | | | 6 | | | | | 6 | | | | |
| AST | (IU/L) | 44.2 | ± | 3.8 |  |  | 60.5 | ± | 32.2 |  |  | 51.4 | ± | 9.6 |  |  |
| ALT | (IU/L) | 16.2 | ± | 2.5 |  |  | 35.3 | ± | 46.5 |  |  | 18.0 | ± | 3.3 |  |  |
| ALP | (IU/L) | 295.4 | ± | 19.9 |  |  | 279.2 | ± | 29.4 |  |  | 306.1 | ± | 33.8 |  |  |
| TP | (g/dL) | 4.56 | ± | 0.11 |  |  | 4.67 | ± | 0.20 |  |  | 4.61 | ± | 0.12 |  |  |
| Alb | (g/dL) | 2.15 | ± | 0.06 |  |  | 2.17 | ± | 0.07 |  |  | 2.16 | ± | 0.07 |  |  |
| A/G |  | 0.89 | ± | 0.02 |  |  | 0.87 | ± | 0.02 |  |  | 0.88 | ± | 0.02 |  |  |
| T-Bil | (mg/dL) | 0.16 | ± | 0.01 |  |  | 0.15 | ± | 0.01 |  |  | 0.15 | ± | 0.02 |  |  |
| UN | (mg/dL) | 21.6 | ± | 3.7 |  |  | 25.5 | ± | 3.6 |  |  | 24.6 | ± | 4.3 |  |  |
| CRE | (mg/dL) | 0.14 | ± | 0.02 |  |  | 0.14 | ± | 0.02 |  |  | 0.13 | ± | 0.01 |  |  |
| Glu | (mg/dL) | 160.7 | ± | 29.4 |  |  | 172.3 | ± | 31.1 |  |  | 191.3 | ± | 19.7 |  |  |
| T-Cho | (mg/dL) | 81.5 | ± | 2.2 |  |  | 79.5 | ± | 8.3 |  |  | 76.2 | ± | 4.0 | # |  |
| TG | (mg/dL) | 22.1 | ± | 10.1 |  |  | 29.5 | ± | 16.6 |  |  | 31.3 | ± | 8.5 |  |  |
| Na | (mEq/L) | 150.1 | ± | 0.6 |  |  | 150.6 | ± | 1.7 |  |  | 150.2 | ± | 0.8 |  |  |
| K | (mEq/L) | 4.76 | ± | 0.07 |  |  | 4.48 | ± | 0.19 | # |  | 4.55 | ± | 0.36 |  |  |
| Cl | (mEq/L) | 115.6 | ± | 1.1 |  |  | 117.0 | ± | 1.8 |  |  | 117.6 | ± | 2.0 |  |  |
| Ca | (mg/dL) | 8.8 | ± | 0.4 |  |  | 8.8 | ± | 0.3 |  |  | 8.8 | ± | 0.2 |  |  |
| IP | (mg/dL) | 5.0 | ± | 0.6 |  |  | 5.5 | ± | 0.5 |  |  | 5.5 | ± | 0.7 |  |  |
| Each value shows mean ± S.D. | | |  |  |  |  |  |  |  |  |  |  |  |  |  |  |
| Significantly different from the control (saline) group at 0.4 mL/body (#: p<0.05 by Steel-Dwass test). | | | | | | | | | | | | |  |  |  |  |
|  |  |  |  |  |  |  |  |  |  |  |  |  |  |  |  |  |

Table S18. Necropsy findings in male mice (28 days after administration)

|  |  |  |  |  |  |  |  |
| --- | --- | --- | --- | --- | --- | --- | --- |
| Group | | |  |  | Control (saline) | Control (culture media) | CellSaic |
| Dose |  |  |  |  | 0.4 mL/body | 0.4 mL/body | 10^7^ cells/0.4 mL/body |
| Number of animals | | |  |  | 6 | 6 | 6 |
| Findings | | |  |  |  |  |  |
|  | Normal | |  |  | 6 | 6 | 1 |
|  | Subcutis | |  |  |  |  |  |
|  |  | Nodule |  |  | 0 | 0 | 5 |
|  |  |  |  |  |  |  |  |

Table S19. Necropsy findings in female mice (28 days after administration)

|  |  |  |  |  |  |  |  |
| --- | --- | --- | --- | --- | --- | --- | --- |
| Group | | |  |  | Control (saline) | Control (culture media) | CellSaic |
| Dose |  |  |  |  | 0.4 mL/body | 0.4 mL/body | 10^7^ cells/0.4 mL/body |
| Number of animals | | |  |  | 6 | 6 | 6 |
| Findings | | |  |  |  |  |  |
|  | Normal | |  |  | 6 | 6 | 1 |
|  | Subcutis | |  |  |  |  |  |
|  |  | Nodule |  |  | 0 | 0 | 5 |
|  |  |  |  |  |  |  |  |

Table S20. Necropsy findings in male mice (56 days after administration)

|  |  |  |  |  |  |  |  |
| --- | --- | --- | --- | --- | --- | --- | --- |
| Group | | |  |  | Control (saline) | Control (culture media) | CellSaic |
| Dose |  |  |  |  | 0.4 mL/body | 0.4 mL/body | 10^7^ cells/0.4 mL/body |
| Number of animals | | |  |  | 6 | 6 | 6 |
| Findings | | |  |  |  |  |  |
|  | Normal | |  |  | 6 | 6 | 0 |
|  | Subcutis (injection site) | | |  |  |  |  |
|  |  | Nodule |  |  | 0 | 0 | 6 |
|  |  |  |  |  |  |  |  |

Table S21. Necropsy findings in female mice (56 days after administration)

|  |  |  |  |  |  |  |  |
| --- | --- | --- | --- | --- | --- | --- | --- |
| Group | | |  |  | Control (saline) | Control (culture media) | CellSaic |
| Dose |  |  |  |  | 0.4 mL/body | 0.4 mL/body | 10^7^ cells/0.4 mL/body |
| Number of animals | | |  |  | 6 | 6 | 6 |
| Findings | | |  |  |  |  |  |
|  | Normal | |  |  | 6 | 6 | 1 |
|  | Subcutis (injection site) | | |  |  |  |  |
|  |  | Nodule |  |  | 0 | 0 | 5 |
|  |  |  |  |  |  |  |  |

Table S22. Organ weights in male mice (28 days after administration)

|  |  |  |  |  |  |  |  |  |  |  |  |  |  |  |  |  |
| --- | --- | --- | --- | --- | --- | --- | --- | --- | --- | --- | --- | --- | --- | --- | --- | --- |
| Group |  | Control (saline) |  |  |  |  | Control (culture media) |  |  |  |  | CellSaic |  |  |  |  |
| Dose |  | 0.4 mL/body |  |  |  |  | 0.4 mL/body |  |  |  |  | 10^7^ cells/0.4 mL/body |  |  |  |  |
| Number of animals |  | 6 | | | | | 6 | | | | | 6 | | | | |
| Body weight | (g) | 22.9 | ± | 1.4 |  |  | 22.5 | ± | 0.8 |  |  | 22.2 | ± | 0.6 |  |  |
| Brain | (g) | 0.47 | ± | 0.01 |  |  | 0.47 | ± | 0.02 |  |  | 0.47 | ± | 0.01 |  |  |
|  | (g%) | 2.08 | ± | 0.13 |  |  | 2.11 | ± | 0.09 |  |  | 2.11 | ± | 0.07 |  |  |
| Salivary glands | (g) | 0.17 | ± | 0.01 |  |  | 0.16 | ± | 0.02 |  |  | 0.16 | ± | 0.02 |  |  |
|  | (g%) | 0.72 | ± | 0.06 |  |  | 0.72 | ± | 0.07 |  |  | 0.71 | ± | 0.09 |  |  |
| Lungs | (g) | 0.16 | ± | 0.02 |  |  | 0.17 | ± | 0.01 |  |  | 0.16 | ± | 0.01 |  |  |
|  | (g%) | 0.69 | ± | 0.10 |  |  | 0.76 | ± | 0.05 |  |  | 0.72 | ± | 0.05 |  |  |
| Heart | (g) | 0.11 | ± | 0.01 |  |  | 0.11 | ± | 0.01 |  |  | 0.11 | ± | 0.01 |  |  |
|  | (g%) | 0.48 | ± | 0.03 |  |  | 0.47 | ± | 0.03 |  |  | 0.50 | ± | 0.03 |  |  |
| Liver | (g) | 1.09 | ± | 0.09 |  |  | 1.07 | ± | 0.07 |  |  | 1.04 | ± | 0.11 |  |  |
|  | (g%) | 4.75 | ± | 0.26 |  |  | 4.76 | ± | 0.32 |  |  | 4.68 | ± | 0.41 |  |  |
| Spleen | (mg) | 15.8 | ± | 4.9 |  |  | 14.6 | ± | 2.2 |  |  | 16.4 | ± | 9.9 |  |  |
|  | (mg%) | 68.2 | ± | 17.7 |  |  | 65.1 | ± | 9.7 |  |  | 73.3 | ± | 41.9 |  |  |
| Kidneys | (g) | 0.35 | ± | 0.03 |  |  | 0.34 | ± | 0.02 |  |  | 0.33 | ± | 0.03 |  |  |
|  | (g%) | 1.52 | ± | 0.08 |  |  | 1.52 | ± | 0.05 |  |  | 1.50 | ± | 0.09 |  |  |
| Adrenals | (mg) | 9.5 | ± | 1.7 |  |  | 9.9 | ± | 2.2 |  |  | 7.7 | ± | 2.2 |  |  |
|  | (mg%) | 41.8 | ± | 8.4 |  |  | 43.8 | ± | 9.6 |  |  | 34.7 | ± | 10.0 |  |  |
| Testes | (g) | 0.15 | ± | 0.01 |  |  | 0.16 | ± | 0.01 |  |  | 0.15 | ± | 0.01 |  |  |
|  | (g%) | 0.66 | ± | 0.07 |  |  | 0.70 | ± | 0.05 |  |  | 0.68 | ± | 0.05 |  |  |
| Epididymides | (g) | 0.08 | ± | 0.01 |  |  | 0.09 | ± | 0.01 | # |  | 0.07 | ± | 0.02 |  |  |
|  | (g%) | 0.33 | ± | 0.02 |  |  | 0.40 | ± | 0.04 | # |  | 0.32 | ± | 0.09 |  |  |
| Each value shows mean ± S.D. | |  |  |  |  |  |  |  |  |  |  |  |  |  |  |  |
| Significantly different from the control (saline) group at 0.4 mL/body (#: p<0.05 by Steel-Dwass test). | | | | | | | | | | |  |  |  |  |  |  |
|  |  |  |  |  |  |  |  |  |  |  |  |  |  |  |  |  |

Table S23. Organ weights in female mice (28 days after administration)

|  |  |  |  |  |  |  |  |  |  |  |  |  |  |  |  |  |
| --- | --- | --- | --- | --- | --- | --- | --- | --- | --- | --- | --- | --- | --- | --- | --- | --- |
| Group |  | Control (saline) |  |  |  |  | Control (culture media) |  |  |  |  | CellSaic |  |  |  |  |
| Dose |  | 0.4 mL/body |  |  |  |  | 0.4 mL/body |  |  |  |  | 10^7^ cells/0.4 mL/body |  |  |  |  |
| Number of animals |  | 6 | | | | | 6 | | | | | 6 | | | | |
| Body weight | (g) | 18.9 | ± | 1.3 |  |  | 18.4 | ± | 1.1 |  |  | 19.0 | ± | 1.7 |  |  |
| Brain | (g) | 0.49 | ± | 0.02 |  |  | 0.47 | ± | 0.02 |  |  | 0.48 | ± | 0.02 |  |  |
|  | (g%) | 2.60 | ± | 0.11 |  |  | 2.58 | ± | 0.14 |  |  | 2.55 | ± | 0.20 |  |  |
| Salivary glands | (g) | 0.10 | ± | 0.01 |  |  | 0.10 | ± | 0.01 |  |  | 0.09 | ± | 0.01 |  |  |
|  | (g%) | 0.52 | ± | 0.06 |  |  | 0.52 | ± | 0.04 |  |  | 0.45 | ± | 0.06 | * |  |
| Lungs | (g) | 0.15 | ± | 0.01 |  |  | 0.15 | ± | 0.02 |  |  | 0.14 | ± | 0.02 |  |  |
|  | (g%) | 0.78 | ± | 0.05 |  |  | 0.79 | ± | 0.12 |  |  | 0.72 | ± | 0.06 |  |  |
| Heart | (g) | 0.09 | ± | 0.01 |  |  | 0.09 | ± | 0.01 |  |  | 0.09 | ± | 0.01 |  |  |
|  | (g%) | 0.48 | ± | 0.02 |  |  | 0.49 | ± | 0.06 |  |  | 0.47 | ± | 0.06 |  |  |
| Liver | (g) | 0.91 | ± | 0.10 |  |  | 0.86 | ± | 0.05 |  |  | 0.87 | ± | 0.16 |  |  |
|  | (g%) | 4.81 | ± | 0.29 |  |  | 4.68 | ± | 0.22 |  |  | 4.52 | ± | 0.47 |  |  |
| Spleen | (mg) | 18.9 | ± | 4.2 |  |  | 17.6 | ± | 4.4 |  |  | 17.3 | ± | 7.5 |  |  |
|  | (mg%) | 99.2 | ± | 18.5 |  |  | 94.9 | ± | 19.3 |  |  | 88.9 | ± | 29.9 |  |  |
| Kidneys | (g) | 0.23 | ± | 0.02 |  |  | 0.22 | ± | 0.01 |  |  | 0.23 | ± | 0.02 |  |  |
|  | (g%) | 1.22 | ± | 0.06 |  |  | 1.20 | ± | 0.06 |  |  | 1.19 | ± | 0.03 |  |  |
| Adrenals | (mg) | 8.1 | ± | 0.8 |  |  | 9.1 | ± | 1.1 |  |  | 7.4 | ± | 1.2 | † |  |
|  | (mg%) | 42.7 | ± | 5.1 |  |  | 49.5 | ± | 5.6 |  |  | 38.8 | ± | 5.9 | † |  |
| Ovaries | (mg) | 11.4 | ± | 1.9 |  |  | 10.6 | ± | 2.8 |  |  | 9.8 | ± | 3.1 |  |  |
|  | (mg%) | 60.6 | ± | 12.3 |  |  | 57.8 | ± | 13.8 |  |  | 51.1 | ± | 13.8 |  |  |
| Uterus | (g) | 0.09 | ± | 0.03 |  |  | 0.08 | ± | 0.03 |  |  | 0.09 | ± | 0.04 |  |  |
|  | (g%) | 0.47 | ± | 0.17 |  |  | 0.44 | ± | 0.14 |  |  | 0.49 | ± | 0.22 |  |  |
| Each value shows mean ± S.D. | |  |  |  |  |  |  |  |  |  |  |  |  |  |  |  |
| Significantly different from the control (saline) group at 0.4 mL/body (*: p<0.05 by Tukey-Kramer test). | | | | | | | | | | |  |  |  |  |  |  |
| Significantly different from the control (culture media) group at 0.4 mL/body (†: p<0.05 by Tukey-Kramer test). | | | | | | | | | | | | |  |  |  |  |
|  |  |  |  |  |  |  |  |  |  |  |  |  |  |  |  |  |

Table S24. Organ weights in male mice ( 56 days after administration)

|  |  |  |  |  |  |  |  |  |  |  |  |  |  |  |  |  |
| --- | --- | --- | --- | --- | --- | --- | --- | --- | --- | --- | --- | --- | --- | --- | --- | --- |
| Group |  | Control (saline) |  |  |  |  | Control (culture media) |  |  |  |  | CellSaic |  |  |  |  |
| Dose |  | 0.4 mL/body |  |  |  |  | 0.4 mL/body |  |  |  |  | 10^7^ cells/0.4 mL/body |  |  |  |  |
| Number of animals |  | 6 | | | | | 6 | | | | | 6 | | | | |
| Body weight | (g) | 24.2 | ± | 0.7 |  |  | 24.7 | ± | 1.1 |  |  | 24.0 | ± | 0.9 |  |  |
| Brain | (g) | 0.48 | ± | 0.01 |  |  | 0.48 | ± | 0.02 |  |  | 0.47 | ± | 0.00 |  |  |
|  | (g%) | 1.97 | ± | 0.07 |  |  | 1.96 | ± | 0.10 |  |  | 1.96 | ± | 0.07 |  |  |
| Salivary glands | (g) | 0.16 | ± | 0.02 |  |  | 0.16 | ± | 0.02 |  |  | 0.16 | ± | 0.01 |  |  |
|  | (g%) | 0.66 | ± | 0.08 |  |  | 0.65 | ± | 0.08 |  |  | 0.65 | ± | 0.04 |  |  |
| Lungs | (g) | 0.17 | ± | 0.01 |  |  | 0.16 | ± | 0.03 |  |  | 0.16 | ± | 0.01 |  |  |
|  | (g%) | 0.69 | ± | 0.02 |  |  | 0.64 | ± | 0.11 |  |  | 0.67 | ± | 0.05 |  |  |
| Heart | (g) | 0.11 | ± | 0.01 |  |  | 0.12 | ± | 0.01 |  |  | 0.11 | ± | 0.01 |  |  |
|  | (g%) | 0.47 | ± | 0.02 |  |  | 0.47 | ± | 0.03 |  |  | 0.47 | ± | 0.04 |  |  |
| Liver | (g) | 1.22 | ± | 0.03 |  |  | 1.23 | ± | 0.11 |  |  | 1.14 | ± | 0.05 | # |  |
|  | (g%) | 5.06 | ± | 0.16 |  |  | 4.98 | ± | 0.30 |  |  | 4.72 | ± | 0.20 |  |  |
| Spleen | (mg) | 15.5 | ± | 1.3 |  |  | 16.1 | ± | 3.1 |  |  | 13.7 | ± | 2.9 |  |  |
|  | (mg%) | 63.9 | ± | 4.0 |  |  | 65.2 | ± | 12.0 |  |  | 56.6 | ± | 10.7 |  |  |
| Kidneys | (g) | 0.36 | ± | 0.02 |  |  | 0.38 | ± | 0.02 |  |  | 0.34 | ± | 0.01 | † |  |
|  | (g%) | 1.48 | ± | 0.08 |  |  | 1.52 | ± | 0.06 |  |  | 1.43 | ± | 0.06 |  |  |
| Adrenals | (mg) | 9.5 | ± | 2.4 |  |  | 8.8 | ± | 3.0 |  |  | 8.1 | ± | 1.5 |  |  |
|  | (mg%) | 39.3 | ± | 9.5 |  |  | 35.6 | ± | 11.3 |  |  | 33.6 | ± | 5.4 |  |  |
| Testes | (g) | 0.17 | ± | 0.01 |  |  | 0.17 | ± | 0.01 |  |  | 0.17 | ± | 0.01 |  |  |
|  | (g%) | 0.68 | ± | 0.04 |  |  | 0.68 | ± | 0.05 |  |  | 0.69 | ± | 0.05 |  |  |
| Epididymides | (g) | 0.08 | ± | 0.00 |  |  | 0.09 | ± | 0.01 |  |  | 0.08 | ± | 0.02 |  |  |
|  | (g%) | 0.34 | ± | 0.01 |  |  | 0.34 | ± | 0.04 |  |  | 0.33 | ± | 0.08 |  |  |
| Each value shows mean ± S.D. | |  |  |  |  |  |  |  |  |  |  |  |  |  |  |  |
| Significantly different from the control (saline) group at 0.4 mL/body (#: p<0.05 by Steel-Dwass test). | | | | | | | | | | |  |  |  |  |  |  |
| Significantly different from the control (culture media) group at 0.4 mL/body (†: p<0.05 by Tukey-Kramer test). | | | | | | | | | | | | |  |  |  |  |
|  |  |  |  |  |  |  |  |  |  |  |  |  |  |  |  |  |

Table S25. Organ weights in female mice (56 days after administration)

|  |  |  |  |  |  |  |  |  |  |  |  |  |  |  |  |  |
| --- | --- | --- | --- | --- | --- | --- | --- | --- | --- | --- | --- | --- | --- | --- | --- | --- |
| Group |  | Control (saline) |  |  |  |  | Control (culture media) |  |  |  |  | CellSaic |  |  |  |  |
| Dose |  | 0.4 mL/body |  |  |  |  | 0.4 mL/body |  |  |  |  | 10^7^ cells/0.4 mL/body |  |  |  |  |
| Number of animals |  | 6 | | | | | 6 | | | | | 6 | | | | |
| Body weight | (g) | 19.1 | ± | 0.4 |  |  | 19.7 | ± | 1.0 |  |  | 18.9 | ± | 0.8 |  |  |
| Brain | (g) | 0.47 | ± | 0.01 |  |  | 0.48 | ± | 0.01 |  |  | 0.49 | ± | 0.01 |  |  |
|  | (g%) | 2.48 | ± | 0.04 |  |  | 2.46 | ± | 0.12 |  |  | 2.60 | ± | 0.11 |  |  |
| Salivary glands | (g) | 0.10 | ± | 0.01 |  |  | 0.10 | ± | 0.01 |  |  | 0.10 | ± | 0.01 |  |  |
|  | (g%) | 0.51 | ± | 0.05 |  |  | 0.49 | ± | 0.05 |  |  | 0.51 | ± | 0.04 |  |  |
| Lungs | (g) | 0.15 | ± | 0.01 |  |  | 0.15 | ± | 0.01 |  |  | 0.15 | ± | 0.02 |  |  |
|  | (g%) | 0.78 | ± | 0.08 |  |  | 0.78 | ± | 0.05 |  |  | 0.80 | ± | 0.08 |  |  |
| Heart | (g) | 0.09 | ± | 0.01 |  |  | 0.09 | ± | 0.01 |  |  | 0.10 | ± | 0.01 |  |  |
|  | (g%) | 0.47 | ± | 0.05 |  |  | 0.47 | ± | 0.04 |  |  | 0.55 | ± | 0.06 | † |  |
| Liver | (g) | 0.87 | ± | 0.05 |  |  | 0.91 | ± | 0.12 |  |  | 0.85 | ± | 0.07 |  |  |
|  | (g%) | 4.52 | ± | 0.20 |  |  | 4.61 | ± | 0.37 |  |  | 4.52 | ± | 0.34 |  |  |
| Spleen | (mg) | 16.4 | ± | 1.4 |  |  | 18.4 | ± | 4.1 |  |  | 17.6 | ± | 3.1 |  |  |
|  | (mg%) | 86.0 | ± | 7.9 |  |  | 93.5 | ± | 20.9 |  |  | 92.9 | ± | 12.4 |  |  |
| Kidneys | (g) | 0.23 | ± | 0.00 |  |  | 0.24 | ± | 0.01 |  |  | 0.23 | ± | 0.01 |  |  |
|  | (g%) | 1.19 | ± | 0.02 |  |  | 1.22 | ± | 0.04 |  |  | 1.22 | ± | 0.04 |  |  |
| Adrenals | (mg) | 7.1 | ± | 1.2 |  |  | 8.8 | ± | 1.1 |  |  | 7.3 | ± | 1.6 |  |  |
|  | (mg%) | 37.2 | ± | 6.4 |  |  | 45.0 | ± | 7.1 |  |  | 38.7 | ± | 8.5 |  |  |
| Ovaries | (mg) | 10.3 | ± | 1.3 |  |  | 12.2 | ± | 2.3 |  |  | 11.8 | ± | 2.5 |  |  |
|  | (mg%) | 54.0 | ± | 6.9 |  |  | 62.0 | ± | 11.1 |  |  | 62.9 | ± | 13.9 |  |  |
| Uterus | (g) | 0.09 | ± | 0.03 |  |  | 0.10 | ± | 0.04 |  |  | 0.09 | ± | 0.04 |  |  |
|  | (g%) | 0.49 | ± | 0.18 |  |  | 0.53 | ± | 0.21 |  |  | 0.47 | ± | 0.16 |  |  |
| Each value shows mean ± S.D. | |  |  |  |  |  |  |  |  |  |  |  |  |  |  |  |
| Significantly different from the control (culture media) group at 0.4 mL/body (†: p<0.05 by Tukey-Kramer test). | | | | | | | | | | | | |  |  |  |  |
|  |  |  |  |  |  |  |  |  |  |  |  |  |  |  |  |  |

Table S26. Histopathological findings in male mice (28 days after administration)

|  |  |  |  |  |  |  |  |  |  |  |  |  |  |  |  |  |  |
| --- | --- | --- | --- | --- | --- | --- | --- | --- | --- | --- | --- | --- | --- | --- | --- | --- | --- |
| Group |  |  | Control (saline) | | | | | Control (culture media) | | | | | CellSaic | | | | |
| Dose | | | 0.4 mL/body | | | | | 0.4 mL/body | | | | | 10^7^ cells/0.4 mL/body | | | | |
| Number of animals | | | 6 | | | | | 6 | | | | | 6 | | | | |
| Grade | | |  | - |  | + |  |  | - |  | + |  |  | - |  | + |  |
| Findings | | |  |  |  |  |  |  |  |  |  |  |  |  |  |  |  |
|  | Injection site | |  |  |  |  |  |  |  |  |  |  |  |  |  |  |  |
|  |  | Residual test substance-like material |  | 6 |  | 0 |  |  | 6 |  | 0 |  |  | 1 |  | 5* |  |
| Grade of residual test substance-like material: -: not recognized, +: recognized. | | | | | | |  |  |  |  |  |  |  |  |  |  |  |
| *:Tumorigenesis was not recognized. | | |  |  |  |  |  |  |  |  |  |  |  |  |  |  |  |
| Examined organs/tissues were the heart, aorta, lung, trachea, liver, gall bladder, pancreas, tongue, sublingual gland, submandibular gland, | | | | | | | | | | | | | | | |  |  |
| esophagus, stomach, duodenum, jejunum, ileum, cecum, colon, rectum, spleen, kidney, urinary bladder, testis, epididymis, seminal vesicle, | | | | | | | | | | | | | | | | |  |
| pituitary, adrenal, thyroid, cerebrum, cerebellum, medulla oblongata, spinal cord, eyeball, Harderian gland, sciatic nerve,rectus femoris muscle, | | | | | | | | | | | | | | | | |  |
| bone (sternum and femur), bone marrow (sternum and femur), and injection site in the control (saline) group and the CellSaic group. | | | | | | | | | | | | | | |  |  |  |
| Only injection site was examined in the control (culture media) group. | | | | |  |  |  |  |  |  |  |  |  |  |  |  |  |
|  |  |  |  |  |  |  |  |  |  |  |  |  |  |  |  |  |  |

Table S27. Histopathological findings in female mice (28 days after administration)

|  |  |  |  |  |  |  |  |  |  |  |  |  |  |  |  |  |  |
| --- | --- | --- | --- | --- | --- | --- | --- | --- | --- | --- | --- | --- | --- | --- | --- | --- | --- |
| Group |  |  | Control (saline) | | | | | Control (culture media) | | | | | CellSaic | | | | |
| Dose | | | 0.4 mL/body | | | | | 0.4 mL/body | | | | | 10^7^ cells/0.4 mL/body | | | | |
| Number of animals | | | 6 | | | | | 6 | | | | | 6 | | | | |
| Grade |  |  |  | - |  | + |  |  | - |  | + |  |  | - |  | + |  |
| Findings | | |  |  |  |  |  |  |  |  |  |  |  |  |  |  |  |
|  | Injection site | |  |  |  |  |  |  |  |  |  |  |  |  |  |  |  |
|  |  | Residual test substance-like material |  | 6 |  | 0 |  |  | 6 |  | 0 |  |  | 1 |  | 5* |  |
| Grade of residual test substance-like material: -: not recognized, +: recognized. | | | | | | |  |  |  |  |  |  |  |  |  |  |  |
| *:Tumorigenesis was not recognized. | | |  |  |  |  |  |  |  |  |  |  |  |  |  |  |  |
| Examined organs/tissues were the heart, aorta, lung, trachea, liver, gall bladder, pancreas, tongue, sublingual gland, submandibular gland, | | | | | | | | | | | | | | | |  |  |
| esophagus, stomach, duodenum, jejunum, ileum, cecum, colon, rectum, spleen, kidney, urinary bladder, ovary, uterus, vargina, | | | | | | | | | | | | | | |  |  |  |
| mammary gland, pituitary, adrenal, thyroid, cerebrum, cerebellum, medulla oblongata, spinal cord, eyeball, Harderian gland, sciatic nerve, | | | | | | | | | | | | | | | |  |  |
| rectus femoris muscle, bone (sternum and femur), bone marrow (sternum and femur), and injection site in the control (saline) group | | | | | | | | | | | | | | |  |  |  |
| and the CellSaic group. | | |  |  |  |  |  |  |  |  |  |  |  |  |  |  |  |
| Only injection site was examined in the control (culture media) group. | | | | |  |  |  |  |  |  |  |  |  |  |  |  |  |
|  |  |  |  |  |  |  |  |  |  |  |  |  |  |  |  |  |  |

Table S28. Histopathological findings in male mice (56 days after administration)

|  |  |  |  |  |  |  |  |  |  |  |  |  |  |  |  |  |  |
| --- | --- | --- | --- | --- | --- | --- | --- | --- | --- | --- | --- | --- | --- | --- | --- | --- | --- |
| Group |  |  | Control (saline) | | | | | Control (culture media) | | | | | CellSaic | | | | |
| Dose | | | 0.4 mL/body | | | | | 0.4 mL/body | | | | | 10^7^ cells/0.4 mL/body | | | | |
| Number of animals | | | 6 | | | | | 6 | | | | | 6 | | | | |
| Grade |  |  | - | ± | + | 2+ | 3+ | - | ± | + | 2+ | 3+ | - | ± | + | 2+ | 3+ |
| Findings | | |  |  |  |  |  |  |  |  |  |  |  |  |  |  |  |
|  | Kidney | |  |  |  |  |  |  |  | NE |  |  |  |  |  |  |  |
|  |  | Cyst, lateral | 5 | 1 | 0 | 0 | 0 |  |  |  |  |  | 6 | 0 | 0 | 0 | 0 |
|  | Testis | |  |  |  |  |  |  |  | NE |  |  |  |  |  |  |  |
|  |  | Dilatation, seminiferous tuble, lateral | 6 | 0 | 0 | 0 | 0 |  |  |  |  |  | 5 | 1 | 0 | 0 | 0 |
|  | Injection site | |  |  |  |  |  |  |  |  |  |  |  |  |  |  |  |
|  |  | Residual test substance-like material | 6 |  | 0 |  |  | 6 |  | 0 |  |  | 0 |  | 6* |  |  |
| Grade of residual test substance-like material: -: not recognized, +: recognized. | | | | | | |  |  |  |  |  |  |  |  |  |  |  |
| Grade of other histopathological findings: -: none, ±: slight, +: mild, 2+: moderate, 3+: marked. | | | | | | | | |  |  |  |  |  |  |  |  |  |
| *:Tumorigenesis was not recognized. | | |  |  |  |  |  |  |  |  |  |  |  |  |  |  |  |
| NE:not examined. | | |  |  |  |  |  |  |  |  |  |  |  |  |  |  |  |
| Examined organs/tissues were the heart, aorta, lung, trachea, liver, gall bladder, pancreas, tongue, sublingual gland, submandibular gland, | | | | | | | | | | | | | | | |  |  |
| esophagus, stomach, duodenum, jejunum, ileum, cecum, colon, rectum, spleen, kidney, urinary bladder, testis, epididymis, seminal vesicle, | | | | | | | | | | | | | | | | |  |
| pituitary, adrenal, thyroid, cerebrum, cerebellum, medulla oblongata, spinal cord, eyeball, Harderian gland, sciatic nerve,rectus femoris muscle, | | | | | | | | | | | | | | | | |  |
| bone (sternum and femur), bone marrow (sternum and femur), and injection site in the control (saline) group and the CellSaic group. | | | | | | | | | | | | | | |  |  |  |
| Only injection site was examined in the control (culture media) group. | | | | |  |  |  |  |  |  |  |  |  |  |  |  |  |
|  |  |  |  |  |  |  |  |  |  |  |  |  |  |  |  |  |  |

Table S29. Histopathological findings in female mice (56 days after administration)

|  |  |  |  |  |  |  |  |  |  |  |  |  |  |  |  |  |  |  |
| --- | --- | --- | --- | --- | --- | --- | --- | --- | --- | --- | --- | --- | --- | --- | --- | --- | --- | --- |
| Group |  |  | Control (saline) | | | | | Control (culture media) | | | | | CellSaic | | | | |  |
| Dose | | | 0.4 mL/body | | | | | 0.4 mL/body | | | | | 10^7^ cells/0.4 mL/body | | | | |  |
| Number of animals | | | 6 | | | | | 6 | | | | | 6 | | | | |  |
| Grade |  |  | - | ± | + | 2+ | 3+ | - | ± | + | 2+ | 3+ | - | ± | + | 2+ | 3+ |  |
| Findings | | |  |  |  |  |  |  |  |  |  |  |  |  |  |  |  |  |
|  | Kidney | |  |  |  |  |  |  |  | NE |  |  |  |  |  |  |  |  |
|  |  | Cyst, lateral | 5 | 1 | 0 | 0 | 0 |  |  |  |  |  | 6 | 0 | 0 | 0 | 0 |  |
|  | Pituitary | |  |  |  |  |  |  |  | NE |  |  |  |  |  |  |  |  |
|  |  | Cyst, anterior lobe | 6 | 0 | 0 | 0 | 0 |  |  |  |  |  | 5 | 1 | 0 | 0 | 0 |  |
|  | Eyeball | |  |  |  |  |  |  |  | NE |  |  |  |  |  |  |  |  |
|  |  | Retinal dysplasia, lateral | 5 | 1 | 0 | 0 | 0 |  |  |  |  |  | 6 | 0 | 0 | 0 | 0 |  |
|  | Injection site | |  |  |  |  |  |  |  |  |  |  |  |  |  |  |  |  |
|  |  | Residual test substance-like material | 6 |  | 0 |  |  | 6 |  | 0 |  |  | 1 |  | 5* |  |  |  |
| Grade of residual test substance-like material: -: not recognized, +: recognized. | | | | | | |  |  |  |  |  |  |  |  |  |  |  |  |
| Grade of other histopathological findings: -: none, ±: slight, +: mild, 2+: moderate, 3+: marked. | | | | | | | | |  |  |  |  |  |  |  |  |  |  |
| *:Tumorigenesis was not recognized. | | |  |  |  |  |  |  |  |  |  |  |  |  |  |  |  |  |
| NE:not examined. | | |  |  |  |  |  |  |  |  |  |  |  |  |  |  |  |  |
| Examined organs/tissues were the heart, aorta, lung, trachea, liver, gall bladder, pancreas, tongue, sublingual gland, submandibular gland, | | | | | | | | | | | | | | | |  |  |  |
| esophagus, stomach, duodenum, jejunum, ileum, cecum, colon, rectum, spleen, kidney, urinary bladder, ovary, uterus, vargina, | | | | | | | | | | | | | | |  |  |  |  |
| mammary gland (except for F01159 and F01162), pituitary, adrenal, thyroid, cerebrum, cerebellum, medulla oblongata, spinal cord, | | | | | | | | | | | | | | |  |  |  |  |
| eyeball, Harderian gland, sciatic nerve, rectus femoris muscle, bone (sternum and femur), bone marrow (sternum and femur), | | | | | | | | | | | | | |  |  |  |  |  |
| and injection site in the control (saline) group and the CellSaic group. | | | | |  |  |  |  |  |  |  |  |  |  |  |  |  |  |
| Only injection site was examined in the control (culture media) group. | | | | |  |  |  |  |  |  |  |  |  |  |  |  |  |  |
|  |  |  |  |  |  |  |  |  |  |  |  |  |  |  |  |  |  |  |
