# Supplementary material for: The Therapeutic Effects of Adipose-Derived Stem Cells and Recombinant Peptide Pieces on Mouse Model of DSS Colitis
Source: Cell Transplant. 2018 Jul 6;27(9):1390–400. doi: 10.1177/0963689718782442 (PMC6168991; doi:10.1177/0963689718782442)
Supplement: Supplementary material [file 782442_Supplemental_material_1_revice.pdf]

Materials and Methods

Safety test

Body weight measurement

Body weight was measured before administration at the day of administration (body weight at the time of grouping), at 1, 4, 7, 11, 14, 18, 21, 25, 28, 32, 35, 39, 42, 46, 49, 53, and 56 days after administration.

Food consumption measurement

Food consumption was measured in periods between the day of administration (feed amount) and day 1 (remaining amount), between day 7 (feed amount) and day 8 (remaining amount), between day 14 (feed amount) and day 15 (remaining amount), between day 21 (feed amount) and day 22 (remaining amount), between day 28 (feed amount) and 29 (remaining amount), between day 35 (feed amount) and 36 (remaining amount), between day 42 (feed amount) and 43 (remaining amount), and between day 49 (feed amount) and day 50 (remaining amount); daily amount was calculated. Food consumption by female mice was calculated by dividing the total consumption amount in each cage by the number of mice in the cage. Food consumption, presented in figures, is the amount remaining on measurement day.

Urinalysis

Urinalysis was performed the day before the day of the necropsy. Fresh urine was collected from all surviving mice, supplied with water in a fasting state, by using urine collection cages; the following tests of Table S1 were

performed. Urine samples were discarded after measurement. If fresh urine could not be collected, it was collected on the following day (necropsy day).

Hematology test

On necropsy day, blood was collected from mice via the abdominal aorta, under anesthesia with isoflurane, using a 1-mL polypropylene disposal syringe (Terumo Corporation, Tokyo, Japan) and a 23G syringe needle (Terumo Corporation) treated with heparin (heparin sodium injection, Japanese Pharmacopoeia compliant, Mochida Pharmaceutical Co., Ltd., Tokyo, Japan). Approximately 150 μL of collected blood was placed in an EDTA-2K-coated tube (BD Microtainer Blood Collection Tube, Becton, Dickinson and Company Japan, Tokyo, Japan) and the following tests of Table S2 were performed. Remaining blood was used in blood biochemistry assessment.

Blood biochemistry test

At the time of blood collection for the hematology test, heparinized blood, collected via the abdominal aorta, was centrifuged (4°C, 2200 × *g*, 15 minutes) using a centrifuge (5417R, Eppendorf Co., Ltd., Tokyo, Japan) and plasma was collected. The following tests of Table S3 were performed using the plasma samples obtained.

Necropsy

After blood was collected for hematology and blood biochemistry tests, mice were sacrificed using exsanguination, and organs and tissues were macroscopically observed.–

Organ weight measurement

At the time of the necropsy, the weights of the following organs were measured for all the surviving mice (electronic balance, AB204, Mettler-Toledo K.K, Tokyo, Japan.): brain (cerebrum, cerebellum, medulla oblongata), salivary gland (submandibular gland, sublingual gland), lung (including bronchus), heart, liver (including gallbladder), spleen, kidney, adrenal gland, male reproductive organs (testis, epididymis), female reproductive organs (ovary, uterus). Paired organs were measured together. First, lung weight, including the trachea, was measured. Then, the lung was inflated with 20 vol% neutral buffered formalin. After that, the trachea was resected and measured, and lung weight was obtained by subtracting the weight of the trachea. Organ weight ratio (relative weight) was calculated by using the weight obtained before the necropsy as reference.

Histopathological examination

At the time of the biopsy, the following organs and tissues were fixed in 20 vol% neutral buffered formalin: Heart, aorta, lung, trachea, liver, gallbladder, pancreas, tongue, salivary gland (sublingual gland, submandibular gland), gastrointestinal tract (esophagus, stomach, duodenum, jejunum, ileum, appendix, colon, rectum), spleen, kidney, urinary bladder, male reproductive organs (testis, epididymis, seminal vesicle), female reproductive organs (ovary, uterus, vagina), mammary gland (female only, when possible), pituitary gland, adrenal gland, thyroid gland, brain (cerebrum, cerebellum, medulla oblongata), spinal cord, eye balls, accessory gland (the Harderian gland), sciatic nerve, rectus femoris muscle, bones and bone marrow (sternum, femur). The sites of administration were also measured. The lung was fixed after inflation with 20 vol% neutral buffered formalin. The testis and epididymis

were fixed in Bouin’s solution for 2 to 3 hours and then fixed in 90 vol% alcohol. Eyeballs were fixed in glutaraldehyde formalin overnight and then again fixed in 20 vol% neutral buffered formalin. Tissues from the control (physiological saline) and CellSaic groups were embedded in paraffin according to standard method; then H&E-stained tissue specimens were prepared and histopathologically examined. Because a specimen-like remnant was found at the administration site in the CellSaic group, the administration sites of mice in the control (culture medium) group were also histopathologically examined. Resected organs and tissues were preserved in 10 vol% neutral buffered formalin.

Statistical methods

Means and standard deviation in each group were calculated for body weight, food consumption, hematology test, blood biochemistry test, and organ weight (including relative weight). Multiple range test was performed among all groups as significance test. Bartlett’s test was performed for equality of variance, and Tukey’s test was performed when the variance was homogeneous. The Steel-Dwass test was performed when variance was not homogeneous. A significance test was performed to compare food consumption between cages of female mice. The significance level was set at 5 %, and the values are presented in two groups of less than 5 % ($p<0.05$) and less than 1 % ($p<0.01$). No significance test was performed for general conditions, urinalysis, necropsy findings, and histopathological examination.

hADSC CellSaic detection in peritoneal cavity

For fluorescent labeling of hADSC CellSaics, DiR (D12731, Thermo Fisher Scientific, Tokyo, Japan) was added to the incubation medium to final concentrations of 3.5 ug/mL during CellSaic formation. DiR-labeled CellSaics were collected at 16-24h after DiR addition and intraperitoneally injected to C57BL/6 mice. Cell number and injection methods were same to the efficacy test described in the materials and methods. DiR-labeled CellSaics were detected by IVIS Imaging System (Perkin elmer) at ex/em 740/790 nm. Skin and peritoneal muscle of each mouse were incised for the detection at intra abdomen.

Results

General conditions

The general conditions of male and female mice are presented in Tables S4 and S5, respectively. Throughout the observation period, none of the female or male mice died or were in a moribund state in all groups. In the control (physiological saline) group, nodules were found at the administration site in all 12 mice (both female and male mice) 2 hours after administration; these nodules disappeared subsequently. No abnormalities were found in general condition of the mice. In the control (culture medium) group, nodules were found at the administration site in all 12 mice (both female and male mice) 2 hours after administration; these nodules disappeared subsequently. No abnormalities were found in the general condition of the mice. In the CellSaic group, nodules were found at the administration site in all 12 mice (both female and male mice) 2 hours after administration. At days 1 to 10, nodules were observed at the administration site, in 3 to 8 male mice and 1 to 4 female mice. The nodules subsequently disappeared, and no abnormalities were detected in the general condition of the mice.

Measurement of body weights

The body weights of male and female mice are presented in Figure S1 and S2, respectively. In female and male mice in all groups, a decrease in body weight (≥0.4 g) was noted at day 1 as compared with that on the day of administration; in female mice in the control (physiological saline) group and CellSaic group, a decrease in body weight (≥0.4 g) was noted at day 56 as compared with that on day 53. On other measurement days, changes were mostly favorable without significant differences among the groups.

Measurement of food consumption

Food consumption by male and female mice is presented in Figure S3 and S4, respectively. In all groups, several male mice showed reduced food consumption between the day of administration and day 1 (<1.5 g). In addition, mean food consumption between the day of administration and day 1 was reduced in 3 female mice in the physiological saline control group and 3 female mice in the culture medium control group. Food consumption by male mice in the CellSaic group, between days 14 and 15, was significantly lower than that in the culture medium control group. Moreover, food consumption by male mice in the CellSaic group, between day 28 and 29, was significantly lower than that in the physiological saline control group. On other measurement days, no significant differences in food consumption were noted for female or male mice in all groups.

Urinalysis

Days 27 and 28

Test results for male and female mice are presented in Tables S6 and S7, respectively. There was nothing noteworthy in female or male mice in all groups.

Days 55 and 56

The test results of male and female mice are presented in Tables S8 and S9 respectively. There was nothing noteworthy in female or male mice in all groups.

Hematology test

Day 28

The test results of male mice and female mice are presented in Tables S10 and S11, respectively. The reticulocyte ratio, in the male mice of the culture medium control group, was significantly lower than that in the physiological saline control group. The eosinophil ratio in the CellSaic group was significantly higher than that in the physiological saline control group. No significant intergroup difference was noted for other measurement parameters in female or male mice.

Day 56

The test results for the male and female mice are presented in Tables S12 and S13, respectively. The reticulocyte count and ratio in the male mice of the CellSaic group were significantly lower than those in the culture medium control group. No significant intergroup difference was noted for other measurement parameters in female or male mice.

Blood biochemistry test

Day 28

The test results for the male and female mice are presented in Tables S14 and S15, respectively. No significant intergroup difference was noted for female or male mice.

Day 56

The test results for male and female mice are presented in Tables S16 and S17, respectively. Potassium levels in female mice of the medium culture control group and total cholesterol in the female mice of the CellSaic group were significantly lower than those in the physiological saline control group. The levels of AST and ALT in a

single female mouse of the culture medium control group were markedly higher than those in other mice in the same group. No significant intergroup differences were noted for other measurement parameters in female or male mice.

Necropsy

Day 28

The findings for the male and female mice are presented in Tables S18 and S19, respectively. No abnormalities were observed in female or male mice in the physiological saline or the medium culture control groups.

Subcutaneous nodules were noted at the administration site of five female and five male mice of the CellSaic group.

Day 56

The findings for the male and female mice are presented in Tables S20 and S21, respectively. No abnormalities were observed in female or male mice of the physiological saline control group or the medium culture control group.

Subcutaneous nodules were noted at the administration site in six male mice and five female mice of the CellSaic group.

Measurement of organ weights

Day 28

The weights of organs in male and female mice are presented in Tables S22 and S23, respectively. The absolute

and relative weights of the epididymides in male mice were significantly higher in the medium culture control group than those in the physiological saline control group. In addition, the relative weights of salivary glands in female mice of the CellSaic group were significantly lower than those in the physiological saline control group. Moreover, the absolute and relative weights of the adrenal gland in female mice of the CellSaic group were significantly lower than those in the culture medium control group. No significant intergroup difference was noted in other measured organs between female or male mice.

Day 56

The weights of organs in male and female mice are presented in Tables S24 and S25, respectively. The absolute weight of the liver in the male mice of the CellSaic group was significantly lower than that in the physiological saline control group. Moreover, the absolute weight of the kidney was significantly lower than that in the control (culture medium) group. In addition, the relative weight of the heart in the female mice of the CellSaic group was significantly higher than that in the culture medium control group. No significant intergroup difference was noted in other measured organs in female or male mice.

Histopathological examination

Day 28

The findings for the male and female mice are presented in Tables S26 and S27, respectively. No abnormalities were observed in female or male mice of the physiological saline control group or the culture medium control group. A specimen-like remnant was found at the administration site of five female and five male mice from the CellSaic group. However, no tumor formation was observed at the site of the specimen-like remnant. A

specimen-like remnant was observed in the mice that had subcutaneous nodules at the site of administration at the time of the necropsy. No abnormalities were noted in other organs or tissues, and there was no tumor formation.

Day 56

The findings for the male and female mice are presented in Tables S28 and S29, respectively. A very mild cyst was observed in the left kidney in one female and one male mouse from the physiological saline control group; mild retinal dysplasia was noted in the right eyeball of a female mouse from this same group. No abnormalities were detected in female or male mice of the culture medium control group. A specimen-like remnant was found at the administration site in six male mice and five female mice of the CellSaic group. However, no tumor formation was observed at the site of this specimen-like remnant. A specimen-like remnant was observed in the mice that had subcutaneous nodules at the administration site at the time of the necropsy. Furthermore, very mild dilation of the seminiferous tubule in the left testis was noted in a male mouse, and a very mild cyst was found in the anterior pituitary of a female mouse. No abnormalities were noted in other organs or tissues, and there was no tumor formation.

Fluorescent labeled hADSC CellSaics were detected in peritoneral cavity

DiR-labeled hADSC Cellsaics were observed as fluorescent spots on abdominal organ surface at day1, 3, 7 and 28 after administration.

Formatted: Font: Bold

Figure Legends

Figure S1 Body weights in male mice.

Control (saline) group; circle, Control (culture media) group: square, CellSaic group; triangle.

Figure S2 Body weights in female mice.

Control (saline) group; circle, Control (culture media) group: square, CellSaic group; triangle.

Figure S3 Food consumption in male mice.

Control (saline) group; circle, Control (culture media) group: square, CellSaic group; triangle. CellSaic group was significantly different from the control (saline) group (*: $P < 0.05$ by Turkey-Kramer test) and the control (culture madia) group (+: $P < 0.05$ by Turkey-Kramer test).

Figure S4 Food consumption in female mice.

Control (saline) group; circle, Control (culture media) group: square, CellSaic group; triangle.

Figure S5 Fluorescent labeled hADSC CellSaic detection by IVIS

DiR-labeled hADSC CellSaics were injected intraperitoneally and fluorescent spots of DiR-labeled hADSC CellSaics were detected by IVIS imaging system at day1, 3, 7 and 28 after administration.

Table S1. Items of urinalysis test

Items	Units	Measurement method	Equipment
Color tone	–	Appearance evaluation	–
pH<br>Protein<br>Glucose<br>Ketone body<br>Bilirubin<br>Occult blood<br>Urobilinogen	–<br>mg/dL<br>mg/dL<br>–<br>-<br>–<br>E.U./dL	Urinalysis test strip	Urine chemistry analyzer* CLINITEK Advantus (Siemens Healthcare Diagnostics K.K.)

*: Visual evaluation was performed when the volume of urine was small.

Table S2. Items of hematological test

Items	Unit	Measurement method	Equipment
Red blood cell count (RBC)	10^4/μL	Sheath flow DC detection method	Automated hematology analyzer XT-2000iV (Sysmex Corporation)
Hemoglobin level (HGB)	g/dL	Sodium lauryl sulfate (SLS) hemoglobin method	
Hematocrit level (HCT)	%	Erythrocyte pulse wave high value detection method	
Platelet count (PLT)	10^4/μL	Sheath flow DC detection method	
Mean corpuscular volume (MCV)	fL	Calculated from RBC and HCT	

Mean corpuscular hemoglobin (MCH)	pg	Calculated from RBC and HGB	
Mean corpuscular hemoglobin concentration (MCHC)	g/dL	Calculated from HCT and HGB	
White blood cell count (WBC)	10^2/μL	Flow cytometry method	
Leukocyte classification	% and 10^2/μL		
Reticulocyte ratio (RET)	%		
Reticulocyte count (RET)	10^4/μL		

Table S3. Irems of blood biochemistry test

Item	Unit	Measurement method	Equipment
AST	IU/L	Malate dehydrogenase-UV assay (JSCC Reference Method)	Biochemistry autoanalyzer AU 480 (Beckman Coulter, Inc.)
ALT	IU/L	Lactate dehydrogenase-UV assay (JSCC Reference Method)	
ALP	IU/L	P-nitrophenyl acetate assay (JSCC Reference Method)	
Total cholesterol (T-Cho)	mg/dL	Cholesterol oxidase-HMMPS method	
Triglyceride (TG)	mg/dL	Glycerol-3-phosphate oxidase-HMMPS method (Glycerol elimination method)	
Total protein (TP)	g/dL	Biuret method	
Albumin (Alb)	g/dL	Bromocresol green method	
A/G	-	Calculated from total protein and albumin level	
Urea nitrogen (UN)	mg/dL	Urease/glutamate dehydrogenase method	
Creatinine (CRE)	mg/dL	Creatininase-HMMPS method	
Total bilirubin (T-Bil)	mg/dL	Biochemical oxygen demand method	
Glucose (Glu)	mg/dL	Hexokinase-glucose 6 phosphate dehydrogenase method	
Inorganic phosphorus (IP)	mg/dL	Purine nucleoside phosphorylase-xanthine dehydrogenase method	

Calcium (Ca)	mg/dL	O cresolphthalein complexion method	
Sodium (Na)	mEq/L	Ion selective electrode method	
Potassium (K)	mEq/L	Ion selective electrode method	
Chlorine (Cl)	mEq/L	Ion selective electrode method	
